# Targeting EGFR Endocytosis and Signaling for Cancer Drug Delivery and Cancer Treatment

**DOI:** 10.3390/cancers18152451

**Published:** 2026-07-30

**Authors:** Xinmei Chen, Zhixiang Wang

**Affiliations:** Department of Medical Genetics, Faculty of Medicine and Dentistry, College of Health Sciences, University of Alberta, Edmonton, AB T6G 2H7, Canada; xinmei6@ualberta.ca

**Keywords:** epidermal growth factor (EGF) receptor (EGFR), cell signaling, endocytosis, cancers, targeted therapeutics, drug delivery, antibody–drug conjugated (ADCs), antibody–nanoparticle conjugates (ANCs)

## Abstract

The epidermal growth factor receptor (EGFR) was the first receptor tyrosine kinase identified soon after v-Src was recognized as a tyrosine kinase. EGFR signaling begins when EGF binds to EGFR at the cell surface, inducing receptor dimerization, activation, and autophosphorylation. The resulting phosphotyrosine sites recruit downstream effectors that activate signaling cascades such as the RAS-RAF-MEK-ERK and PI3K-Akt pathways, thereby regulating cell growth, proliferation, and survival. EGF binding also promotes EGFR endocytosis, which can direct the receptor to lysosomal degradation. Aberrant EGFR activity is associated with many cancers, and the receptor has been therapeutically targeted using small-molecule tyrosine kinase inhibitors (TKIs) and monoclonal antibodies (mAbs). Furthermore, EGFR endocytosis has been exploited for the targeted delivery of anticancer agents into EGFR-expressing cancer cells through antibody–drug conjugates (ADCs) and antibody–nanoparticle conjugates (ANCs). Although ADCs and ANCs both utilize mAbs as homing mechanisms to recognize cancer-associated antigens, they further harness EGFR endocytosis to deliver therapeutic payloads directly into target cells. In this review, we briefly discuss EGFR structure, activation, signaling, and endocytosis, as well as the mechanisms underlying EGFR function in cancer development. We then focus on current advances and future perspectives in using EGFR endocytosis pathways to improve targeted cancer drug delivery and therapy, particularly in the context of ANCs.

## Simple Summary

Epidermal growth factor receptor (EGFR) signaling drives critical cell processes, and its dysfunction is linked to cancer development, prompting the use of targeted inhibitors and antibodies. By leveraging EGFR endocytosis, advanced strategies like antibody–drug conjugates (ADCs) and antibody–nanoparticle conjugates (ANCs) can deliver therapeutic payloads directly into cancer cells. This review explores these mechanisms and discusses advancements in utilizing EGFR internalization pathways to enhance targeted cancer therapies.

## 1. Introduction

The epidermal growth factor receptor (EGFR) was identified shortly after the discovery of v-src as a tyrosine kinase [1,2]. The identification of EGFR phosphorylation provided fundamental insights into how cells receive external signals and translate them into internal responses. EGFR is widely regarded as the prototypical receptor tyrosine kinase (RTK) because it was the first of its kind to be discovered, serving as the primary model for understanding RTK structure, cellular signaling, endocytosis, and targeted cancer therapy [3,4,5].

It is now well established that EGFR (ErbB1), together with ErbB2 (HER2), ErbB3 (HER3), and ErbB4 (HER4), constitutes a subfamily of RTKs termed the EGFR or ErbB family. The EGFR signaling pathway is a complex cellular communication system initiated by the binding of an extracellular ligand, such as EGF, to EGFR on the cell surface. This binding event induces receptor dimerization, leading to kinase activation and autophosphorylation, which creates docking sites for downstream signaling proteins. These proteins subsequently trigger various intracellular cascades, such as the RAS-RAF-MEK-ERK and PI3K-Akt pathways, to regulate cell growth, proliferation, and survival. Concurrently, the binding of EGF to EGFR induces receptor endocytosis, which targets EGFR for trafficking and eventual degradation in lysosomes. Aberrant EGFR activity is linked to the development of numerous malignancies, making the receptor a primary target for cancer treatment [4,5,6,7,8,9,10].

EGFR was the first human RTK shown to possess a viral oncogene counterpart, v-ErbB, establishing a pivotal link between RTK alterations and uncontrolled malignant cell growth [11]. Aberrant EGFR signaling promotes tumor progression and has been implicated in many cancers, including non-small cell lung cancer (NSCLC), glioblastoma multiforme (GBM), head and neck squamous cell carcinoma (HNSCC), colorectal cancer (CRC), pancreatic adenocarcinoma, breast cancer (BC), gastrointestinal and genitourinary cancers, ovarian cancer, and cutaneous melanoma [4,12,13]. Although underlying EGFR alterations vary across distinct cancer types, they commonly hijack pathways controlling cell survival, proliferation, and metabolism.

Accordingly, targeting EGFR has become a cornerstone of precision oncology, as EGFR overexpression or mutation drives uncontrolled proliferation in many solid tumors. Early therapeutic strategies aimed to block downstream mitogenic signaling using monoclonal antibodies (mAbs) and tyrosine kinase inhibitors (TKIs). More recent approaches include antibody–drug conjugates (ADCs) and antibody–nanoparticle conjugates (ANCs), which exploit EGFR endocytosis pathways to deliver anticancer agents selectively into EGFR-expressing cancer cells [14,15,16,17,18]. In this review, we briefly discuss EGFR structure, activation, signaling, and endocytosis, as well as the mechanisms underlying EGFR function in cancer development. We then focus on current progress and future perspectives in exploiting EGFR endocytosis pathways to improve targeted cancer drug delivery and therapy, particularly in the context of ANCs.

## 2. EGFR Structure, Activation, and Signaling

**Figure 1:**
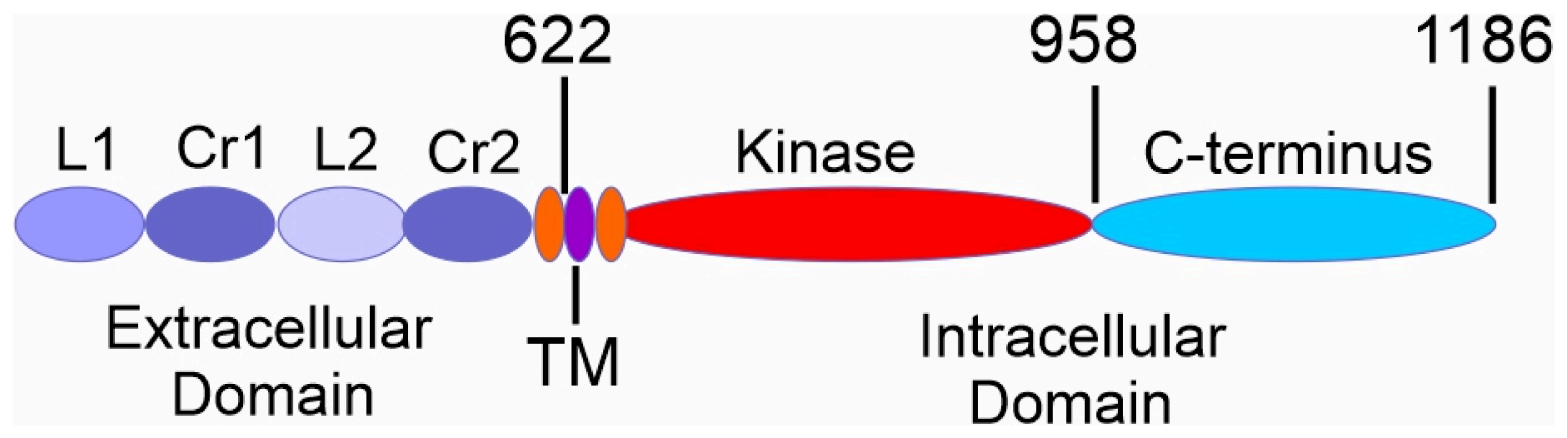
The linear domain structure of EGFR. TM: transmembrane domain.

EGFR is synthesized as a 1210 residue precursor that is cleaved at the N-terminal to result in the mature 1186 residue transmembrane receptor [19]. The mature EGFR is a single-chain transmembrane receptor with an extracellular domain for ligand binding, a transmembrane helix, an intracellular tyrosine kinase (TK) domain, and a C-terminal regulatory domain [3,4,20] (Figure 1).

The extracellular portion of EGFR consists of 621 amino acids and is divided into four domains: I (amino acids 1–133, exons 1–4), II (amino acids 134–312, exons 5–7), III (amino acids 313–445, exons 8–12), and IV (amino acids 446–621, exons 13–16). Domains I (L1) and III (L2) are leucine-rich segments involved in binding ligands. Domain II (Cr1) mediates homo- or hetero-dimerization with similar domains from other family members, while domain IV (Cr2) can form disulfide bonds with domain II and connects to the transmembrane (TM) domain. Notably, domains II and IV are cysteine-rich but do not directly interact with ligands [3,4]. The TM domain itself comprises 23 hydrophobic amino acids, forming a single membrane-spanning segment that secures the receptor within the membrane [21]. Research suggests the EGFR TM domain is involved in dimerization, with the N-terminal region of these helices potentially making contact during this process [22].

The intracellular domain comprises 542 amino acids and consists of the flexible juxtamembrane segment (approximately 40 amino acids), the tyrosine kinase domain (residues 690–953), and the C-terminal regulatory region (residues 958–1186) [3,4]. Structurally, the tyrosine kinase domain is divided into an N-lobe, predominantly formed by β-sheets, and a C-lobe, primarily composed of α-helices, with the ATP-binding site situated between the two lobes [23]. Transautophosphorylation is facilitated by the interaction between the N-lobe of one receptor and the C-lobe of another [24]. The C-terminal tail contains essential tyrosine residues that undergo phosphorylation, thereby serving as docking sites for various signaling molecules [3,20].

EGFR phosphorylation is mostly induced by ligand binding. The EGFR ligand family currently comprises 7 members: EGF, transforming growth factor-α (TGF-α), amphiregulin (AREG), epiregulin (EREG), betacellulin (BTC), heparin-binding EGF-like growth factor (HB-EGF), and EPGN. These ligands bind EGFR and regulate cellular processes such as proliferation, differentiation, and survival. They can also produce distinct downstream responses by stabilizing different EGFR dimmer conformations. EGF, TGF-α, HB-EGF and BTC are high-affinity ligands, whereas AREG, EREG, and EPGN are low-affinity ligand [25,26,27]. EGF is the most common ligand will be the one discussed in this review.

Binding of EGF to EGFR induces the dimerization of EGFR, The activation of EGFR kinase, and trans-autophosphorylation of the C-terminus of two receptor monomers. The EGFR C-terminal tails contain several tyrosine residues that undergo autophosphorylation upon activation of the receptor and serve as docking sites for effector proteins that transmit the signal further downstream. The main tyrosine phosphorylation residues in EGFR tail are Y974, Y992, Y1068, Y1086, Y1148, and Y1173, which serve as docking sites for downstream signaling proteins. Additionally, Y1045 is involved in recruiting the CBL ubiquitin ligase for signal downregulation [5,28,29,30].

Research indicates that two EGF molecules pair with two EGFR molecules to form a complex [31,32]. While ligand-induced dimerization is crucial, some studies suggest that EGFRs can exist as dimers even without ligands, supporting the allosteric activation (rotation model), where either a single ligand or mutations may activate preformed dimers [33]. Additionally, certain amino acid residues in EGFR can be phosphorylated by other kinases, such as non-receptor tyrosine kinases like Src, and serine/threonine kinases including p38, Erk5 (S1046/1047), and PKC (T654). For instance, phosphorylation at Y845 plays a significant role in both cancerous and normal cells [34]. Activation of PKC by phorbol esters induce transient internalization without degradation of unoccupied EGFR [35,36]. On the other hand, p38 MAPK directly drives a specialized, “non-canonical” form of clathrin-mediated endocytosis, which will be discussed in detail later [37].

**Figure 2:**
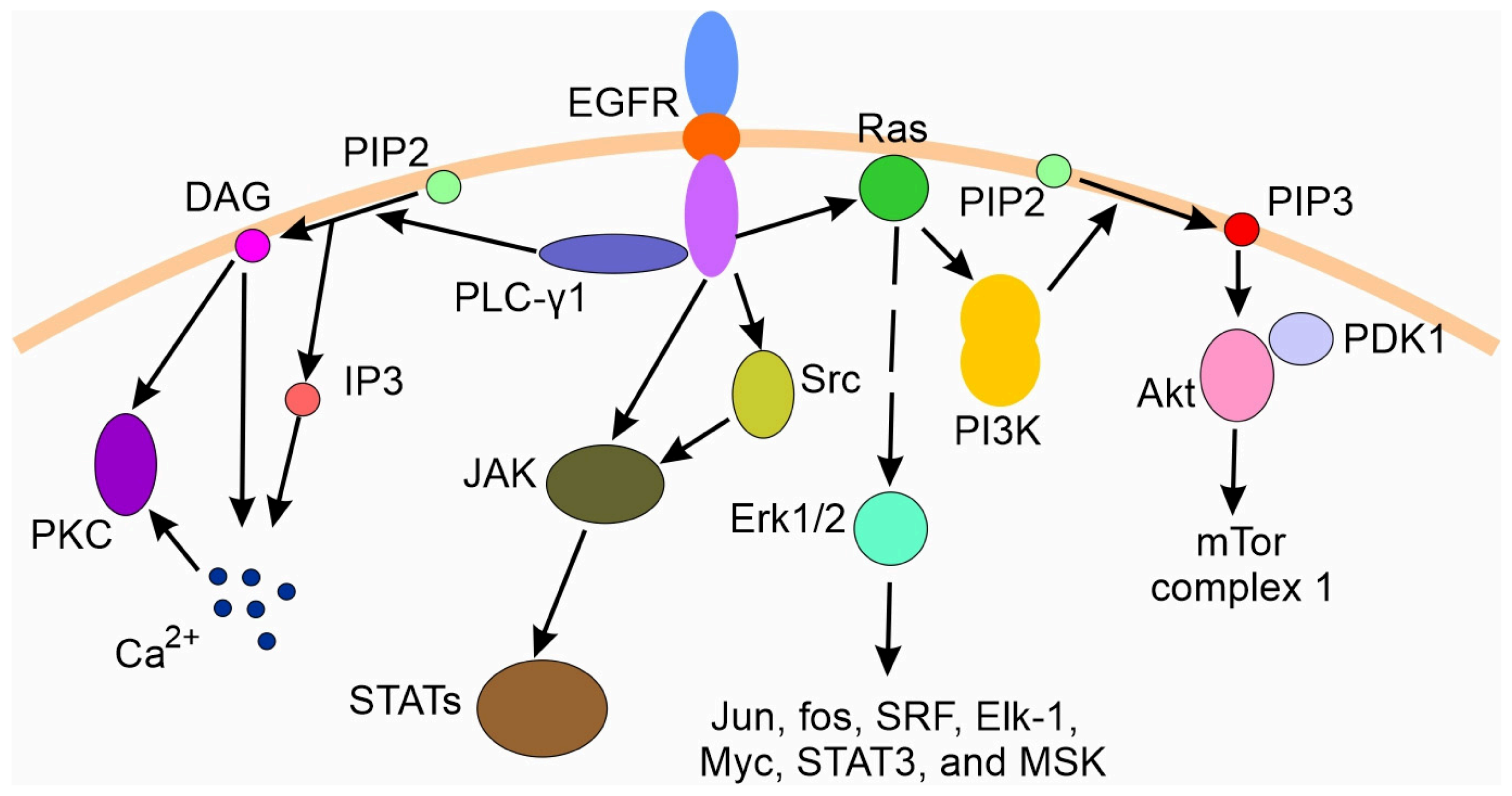
Major signaling pathways mediated by EGFR.

Activated EGFR orchestrates several key signaling cascades, modulating essential cellular functions and leading to significant physiological effects. The principal pathways regulated by EGFR include Ras-ERK, PI3K-Akt, PLC-γ1, and Src signaling pathways (Figure 2) [4,5].

The RAS/RAF/MEK/ERK signaling pathway is a crucial MAPK cascade that regulates cell development, differentiation, proliferation, and death. Isoforms of RAS, RAF, MEK, and ERK vary in their functions and cancer-causing potential. Ras-Erk pathways influence cell behavior by activating transcription factors in the nucleus either directly or indirectly via cytosolic substrates like RSK and MNK. Key transcription factors involved include Jun, Fos, SRF, Elk-1, Myc, STAT3, and MSK [38] (Figure 2).

Activated EGFR triggers PI3K either directly via p85α or indirectly through Ras [39,40]. PI3K converts Phosphatidylinositol 4,5-bisphosphate (PIP2) to Phosphatidylinositol 3,4,5-trisphosphate (PIP3), which is reversed by PTEN as a negative regulator. PIP2 is a structural membrane lipid. When broken down by enzymes, it creates secondary chemical signals (IP_3_ and DAG) that trigger the release of calcium within the cell. PIP3 recruits proteins like Akt to the membrane via PH domains, activating Akt and promoting cell survival—especially in ErbB2/ErbB3 heterodimers linked to cancer. Akt, regulated by phosphorylation at Thr308 (PDK1) and Ser473 (mTORC2), controls metabolism, growth, and apoptosis, with dysfunction associated with diseases such as cancer and diabetes [41]. Akt phosphorylates targets including FoxO, BAD, GSK3, and TSC2, leading to mTORC1 activation. mTORC1 then phosphorylates S6K1 and 4EBP1, enhancing protein synthesis and inhibiting apoptosis [39,42,43] (Figure 2).

The activation of PLC-γ1 by EGFR regulates a spectrum of cellular processes, including proliferation, differentiation, receptor endocytosis, motility, membrane ruffle formation, and branching tubulogenesis [44,45,46,47,48,49,50,51]. Elevated expression and activity of PLC-γ1 have been implicated in the pathogenesis of breast and prostate cancers, with PLC-γ1 activity specifically associated with cancer cell invasion [52,53]. PLC-γ1, a 145 kD protein, contains two SH2 domains, one SH3 domain, and two pleckstrin homology (PH) domains. This enzyme catalyzes the hydrolysis of phosphatidylinositol-4,5-bisphosphate (PtdIns-4,5-P2), generating inositol-1,4,5-triphosphate (IP3) and diacylglycerol (DAG). These second messengers, IP3 and DAG, mediate intracellular Ca^2+^ release and activate protein kinase C (PKC), respectively [44]. In vivo, PLC-γ1 forms complexes with EGFR via SH2 domain interactions [54,55,56,57,58], which leads to tyrosine phosphorylation and increased enzymatic function [59,60,61]. Recent findings demonstrate that PLC-γ1 is extensively involved in cell signaling, with most novel protein interactions engaging its SH3 domain. EGF promotes SH3-mediated associations with PLD2 as well as direct interaction with Akt. The SH3 domain also functions as a guanine nucleotide exchange factor (GEF) for PIKE, dynamin, and Rac1. Activated PLC-γ1 subsequently impacts cell mitogenesis and migration (Figure 2).

EGFR can activate STATs either through direct binding and phosphorylation or indirectly via c-Src. This activation may occur through cytokine (IL-6), growth factor (EGF), or non-receptor tyrosine kinase (Src) signaling pathways. While JAK is not essential for EGFR’s direct activation of STATs, it enhances maximal STAT activation by facilitating Src-mediated phosphorylation. Grb2 inhibits STAT-driven EGFR signaling by preventing STAT binding to EGFR, whereas SOCS reduces STAT activation by binding to JAK and blocking Src’s influence. Once activated, STATs form dimers, translocate to the nucleus, and drive gene transcription that supports proliferation, differentiation, and survival [30,62]. The Src kinase family includes ten membrane-associated, non-receptor tyrosine kinases: Src, Frk, Lck, Lyn, Blk, Hck, Fyn, Yrk, Fgr, and Yes. These kinases are crucial in regulating cellular processes such as proliferation, differentiation, apoptosis, migration, and metabolism. Src signaling interacts with pathways like PI3K and STAT and is particularly prominent in cancers with elevated EGFR expression, enhancing EGFR signaling through Src-dependent phosphorylation and forming complexes with EGFR. In addition to FAK, PI3K and STAT3 are both substrates for c-Src. Although Src has been tied to cancer progression, its exact function remains uncertain. Src operates independently of RTK signaling but can interface with RTKs—including EGFR—to potentially amplify EGFR activity and contribute to resistance against EGFR-targeted therapies (Figure 2).

## 3. EGFR Endocytosis and Its Regulation

EGFR endocytosis is a multiple step process and generally consists of two stages: (1) internalization, which covers the steps from targeting membrane receptors to the specific subdomain of membrane to the complete entry of the receptor into the cell, and (2) intracellular trafficking, which covers the steps after the receptors enter the cell [63].

Under physiological conditions, EGFR undergoes low level and slow endocytosis without binding ligand. However, ligand binding triggers rapid and massive endocytosis of EGFR. Ligand binding accelerates EGFR endocytosis by up to 10-fold, shifting it from a slow, constitutive housekeeping cycle to a rapid, high-capacity clearance mechanism. While unliganded EGFR relies on baseline membrane turnover, ligand binding triggers active molecular changes—specifically receptor dimerization, kinase activation, and multi-ubiquitination—that force the receptor into highly efficient endocytic pathways [64,65].

Endocytic pathways are generally classified as either clathrin-dependent or clathrin-independent. EGFR endocytosis utilizes both clathrin-mediated endocytosis (CME) pathway and non-clathrin-mediated endocytosis (NCE) pathway.

### 3.1. Ligand-Induced Canonic Endocytosis of EGFR

Ligand-induced EGFR endocytosis mostly follows CME under physiological concentration of EGF (1–10 ng/mL). In this pathway, EGFRs are recruited into developing clathrin-coated pits, together with clathrin, adaptor proteins and accessory proteins. Coated pits then form coated vesicles. Following the pinch off coated vesicles from the plasma membrane into the cytoplasm, EGFR completes its internalization process. In the following intracellular trafficking process, EGFR first entered into early sorting endosomes. Some EGFR recycle back to the plasm membrane via recycling endosome; others travel to the late endosomes and continue to lysosomes. In this stage, EGFR dissociated with its bound ligand and gradually degraded [63,66,67]. Cbl plays a prominent role in EGFR lysosome targeting and downregulation by virtue of its E3 ubiquitin ligase activity [68]. Upon EGF induction, Cbl is recruited to activated EGFR, associating directly with phosphotyrosine 1045 (pY1045) on EGFR [69]. Cbl can also associate indirectly with activated EGFR through the adaptor protein Grb2, which binds both Cbl and pY1068 or pY1086 on EGFR [70]. Following binding, Cbl is phosphorylated by EGFR, allowing recruitment of ubiquitin-loaded E2 proteins to its RING finger and subsequent ligation of monomeric ubiquitin at multiple sites along the receptor [69,71,72,73]. The event of multiple monoubiquitination, termed “multiubiquitination,” is thought to be a major determinant in EGFR lysosome targeting and downregulation [74]. However, Cbl’s role in EGFR endocytosis is controversial [75,76]. In addition to Cbl, FBXW7 is another E3 ligase that can target EGFR for degradation [77].

It is known that EGF concentration is at 1–2 ng/mL in most mammalian tissues and tumors, CME has been considered as the major pathway for ligand induced endocytosis [63,66,67,78].

Ligand induced EGFR endocytosis may also go through non-clathrin-mediated endocytosis (NCE). EGFR NCE is a specialized internalization pathway activated primarily at high concentrations of EGF (typically 20–100 ng/mL). While low doses of EGF trigger the canonical clathrin-mediated endocytosis (CME) to promote receptor recycling and sustained signaling, the NCE pathway operates as a critical negative feedback mechanism. It downregulates EGFR activity by preferentially targeting the internalized receptors to lysosomes for degradation, thereby protecting cells from overstimulation [65,79,80,81,82]. Higher EGF concentrations (10–100 ng/mL) are present in fluids such as urine, milk, and saliva [83,84,85] and in response to local conditions such as wound healing [86,87]. The reliance on NCE can shift depending on the cellular context. It is shown that during mitosis, the CME pathway is completely suppressed. Consequently, mitotic cells route EGFR exclusively through CBL-mediated NCE, even when exposed to low concentrations of EGF [82,88].

Although the regulatory mechanisms of ligand-induced EGFR endocytosis are still not completely defined, large amounts of evidence have indicated that many elements play important regulatory roles in this process, including receptor dimerization, tyrosine kinase activation, phosphorylation of receptor and downstream proteins, receptor ubiquitination, and receptor intrinsic endocytic codes [63,88,89,90].

### 3.2. Non-Ligand Induced Non-Canonical Endocytosis of EGFR

EGFR may also undergo CME in the absence of ligand binding. p38 mitogen-activated protein kinase (p38)-mediated EGFR endocytosis is a non-canonical, ligand-independent mechanism where environmental stress or cellular crosstalk forces EGFR to internalize. Unlike canonical endocytosis triggered by EGF binding, this stress-driven pathway reroutes receptor traffic to alter cell survival outcomes [91,92,93]. Cellular stressors including UV light, chemotherapeutics like cisplatin, and inflammatory cytokines like TNF-α strongly activate the p38 pathway. Activated p38 then directly phosphorylates specific serine and threonine residues located on the C-terminal tail of EGFR, primarily targeting Ser-1015, Ser-1046/1047, and Thr-669. This phosphorylation induces a structural shift, allowing the receptor’s dileucine motif to interact directly with the σ2 subunit of the clathrin adaptor protein AP2. p38 also phosphorylates downstream endocytic adaptors like Eps15. The unliganded EGFR monomers are subsequently internalized into the cell via clathrin-coated pits [37,67,92,93,94,95].

The downstream path of the internalized receptor depends entirely on the nature of the stimulus. Under transient stress (Cytokines) EGFR internalizes but rapidly recycles back to the plasma membrane as functional receptors, acting as a feedback system to clear unliganded receptors during low-ligand signaling [37,93]. Under sustained stress (UV/Chemotherapy) EGFR becomes arrested inside Rab5-containing early endosomes. This compartmentalization sequesters the receptor away from the cell surface, shutting down pro-survival and proliferative signaling axes (like AKT/ERK) to facilitate stress-induced apoptosis [91,93,96].

EGFR endocytosis is heavily exploited in precision oncology to either modulate the effects of other cancer therapeutics or achieve targeted drug delivery, which will be discussed in the following up sections [67,96,97,98].

Because many aggressive cancers (such as non-small cell lung cancer, glioblastoma, and colorectal cancers) overexpress EGFR, the receptor acts as a specific “portal” to selectively internalize cytotoxic payloads into malignant cells while sparing healthy tissue. By attaching payloads to antibodies, peptides, or nanoparticles that bind EGFR, bioengineers hijack the cell’s natural internalization machinery to import therapies directly into the cytosol or lysosomes [99,100].

### 3.3. Alternative Fates for Endocytosed EGFR

Besides lysosomal degradation and recycling back to the plasma membrane, EGFR may also traffic to other subcellular locations. Although it is controversial, many studies indicate that EGFR translocate to the nucleus to regulate gene expression [101,102,103]. Nuclear EGFR primarily localizes to the inner nuclear membrane and the nucleoplasm, where it accumulates as a full-length, phosphorylated protein. Once inside these subnuclear locations, EGFR pivots from its traditional plasma membrane role to function directly as a transcriptional co-activator, tyrosine kinase, and regulator of DNA replication and repair [101,102,103,104,105,106,107].

Although the mechanism remains incompletely defined, current evidence indicates that EGFR translocation from the cell surface to the nucleus occurs through a multistep retrograde transport pathway. Rather than dissociating from membranes, full-length EGFR moves through intracellular membrane compartments to reach the nuclear boundary [63,97,108]. Nuclear-destined EGFR is internalized through clathrin-dependent endocytosis [108]. EGF stimulation induces receptor dimerization and internalization into endocytic vesicles. EGFR then undergoes COPI-mediated retrograde trafficking through the Golgi apparatus to the endoplasmic reticulum (ER) [109]. At the ER, EGFR is transferred from the outer nuclear membrane to the inner nuclear membrane through interactions involving importin β and the nuclear pore complex. The soluble nuclear transport factor importin β1 recognizes the specific Nuclear Localization Signal on EGFR’s cytosolic tail. Importin β1 chaperones the membrane-embedded receptor through the nuclear pore complex (NPC), pulling it securely into the Inner Nuclear Membrane (INM). Once at the inner nuclear membrane, EGFR interacts with Sec61, which facilitates its extraction from the membrane and releases into the nucleus [110,111].

EGFR can also translocate directly into the mitochondria. This translocation is also through CME in response to high-concentration EGF stimulation or cellular stress (such as chemotherapy, staurosporine, or tyrosine kinase inhibitors). Unlike nuclear translocation which utilizes an NLS, mitochondrial import requires a distinct, hydrophobic structural sequence within EGFR that functions as a Mitochondrial Localization Signal. The tumor suppressor Tid1-S physically interacts with internalized EGFR in the cytosol. Tid1-S acts as the primary vehicle that guides the receptor to the translocases of the outer/inner mitochondrial membranes for insertion. EGFR often partners with the tyrosine kinase c-Src. Both kinases translocate to the mitochondria with identical kinetics, maintaining a complex even inside the organelle [112,113,114].

Once inside the mitochondria, EGFR physically interacts with metabolic and structural proteins to rewrite cellular bioenergetics. The major functions include inducing mitochondrial fission and metastasis, inhibiting apoptosis, and metabolic overdrive via lipids [112,113,115,116]. Mitochondria EGFR acts as an alternative non-canonical mechanism that tumor cells use to reprogram metabolism, survive apoptotic stress, and drive metastasis [117].

However, alternative fates for activated EGFRs are emerging, including traffic to both the nucleus and the mitochondria. Nuclear EGFR, that is reported to depend on the nuclear localization sequence within the EGFR juxtamembrane region that interacts with importin-β, has been proposed to act as a transcriptional regulator and modulator of DNA repair through interaction with DNA-dependent protein kinase (DNA-PK).

### 3.4. Regulation of EGFR Signaling by Endocytosis

Endocytosis is essential for cells to perceive extracellular signals and transduce them in a temporally and spatially controlled fashion, directly influencing not only the duration and intensity of the signaling output, but also their correct location. It is well established that the endocytosis of EGFR from the plasma membrane to lysosomes results in the degradation of the receptor, which can attenuate receptor signaling and may even be conceived of as a tumor suppressor pathway [118]. On the other hand, accumulated evidence suggests that the internalized EGF-EGFR complex may maintain its ability to generate cell signaling from endosomes [63,118,119,120,121].

Endocytosis has been recognized as the most significant pathway to downregulate EGFR by removing the receptor from the cell surface for degradation in lysosomes [122]. This downregulation of EGFR is a complicated and tightly regulated process. During this process the EGFR-containing internalized vesicles mature into multivesicular bodies (MVBs), which then fuse with lysosomes to allow degradation of their content [123,124].

The role of endocytosis in the downregulation of EGFR signaling is well illustrated by the findings that the inhibition of EGFR endocytosis frequently leads to cancer [125]. The best characterized EGFR mutant with impaired endocytosis is EGFRvIII. EGFRvIII has been implicated in many types of tumors [126,127,128]. EGFRvIII was shown to transform fibroblasts and to enhance the proliferation and/or tumorigenicity of cells both in vivo and in vitro [129,130,131]. Constitutive activity may be important for tumorigenicity, but impaired downregulation certainly enhances the effect [132].

On the other hand, it is now well accepted that internalized EGFR continues to signal form intracellular locations, primarily endosomes [63]. The early evidence to support signaling from endosomes has been reported since middle 1980s. These studies showed that internalized EGFR is autophosphorylated and catalytically active [133,134,135]. Various signaling molecules that regulate Ras activity, including Grb2, SHC, Sos and GAP, are co-internalized with EGFR into endosomes and remain associated with the receptor in endosomes [136,137,138,139].

The major evidence supporting endosomal EGFR signaling came from endocytosis inhibition experiments. These experiments have yielded mixed results regarding what signaling pathways activated by endosomal EGFR and the physiological relevance of EGFR signaling from endosomes [63,140,141]. In addition, a novel system was established to allow the specific activation of endosome-associated EGFR without the initial activation at the plasma membrane and without disrupting the overall endocytosis pathway. It is shown that the specific endosomal EGFR activation leads to strong, but distinct activation of downstream signaling pathways [120,121]. Thus, block EGFR endocytosis will sustain EGFR signaling from the plasma membrane but inhibit its signaling from other intracellular locations including endosomes, mitochondria, and nucleus.

## 4. Mechanisms Underlying EGFR Functions in Cancer

**Figure 3:**
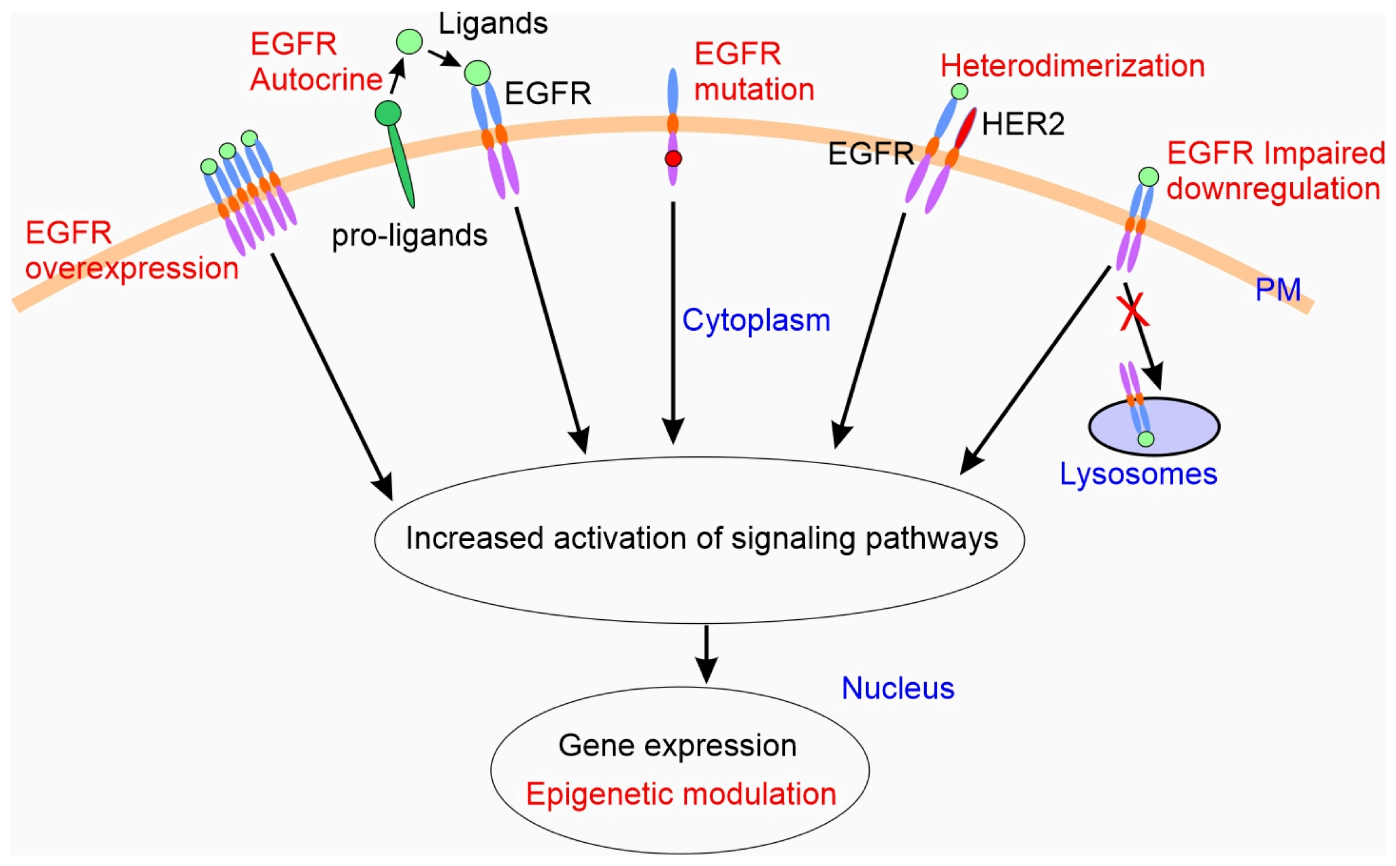
Mechanisms underlying EGFR functions in cancer.

EGFR drives cancer through multiple mechanisms including overexpression, activating mutations (e.g., exon 19 del, L858R), ligand amplification (autocrine loops), crosstalk with other receptors, impaired downregulation, downstream pathway hyperactivation (MAPK, PI3K/Akt), nuclear signaling, and epigenetic modulation. All these mechanisms lead to uncontrolled cell growth, survival, and proliferation, often by keeping signaling pathways constantly “on” even without growth factors [5,142,143] (Figure 3).

### 4.1. EGFR Overexpression and Cancer

While normal EGFR regulates cell growth, the overexpression of EGFR is a critical hallmark in many human cancers, acting as a primary driver for tumor growth, survival, and metastasis. While normal cells contain between 40,000 and 100,000 EGFR, cancer cells can overexpress more than 1 million receptors per cell.

Overexpression can stem from gene amplification or increased gene expression [144], leading to more protein production. An increase in the copy number of the EGFR gene is a frequent cause of protein overabundance and cancer development, especially in HNSCC (80% to 100% of cases) [145] and GBM (more than 50% of cases) [146]. The low-oxygen environment (hypoxia) common in the core of solid tumors can trigger the activation of HIF-2α, which increases the translation of EGFR mRNA into protein without requiring a genetic mutation [144].

At normal levels, EGFR is structurally locked in an inactive, tethered conformation to prevent accidental firing. When EGFR is overexpressed, the local concentration on the plasma membrane skyrockets. The laws of mass action force these tethered molecules into constant physical collisions. The kinetic energy of these crowded, repetitive impacts destabilizes the weak intramolecular tether, causing the receptors to spontaneously unfold into the “extended” (active) conformation without needing a ligand [147,148].

Overexpressed EGFR hyperactivates downstream pathways such as RAS-RAF-MEK-ERK and PI3K-AKT-mTOR, which provide several survival advantages to tumor cells including uncontrolled proliferation, metastasis, angiogenesis, and immune escape. Overexpression of EGFR has been linked to many types of cancers with significant clinical implications including HNSCC, NSCLC, GBM, BC, and CRC [149] (Table 1). This excess signaling promotes aggressive tumor behavior includes larger tumors, poor differentiation, and increased metastasis, but also makes them targets for EGFR-targeted therapies. While a target, tumors often develop resistance through new mutations or activating other pathways [4,142,143].

### 4.2. EGFR Activating Mutations and Cancers

Activating mutations in EGFR gene are primary oncogenic drivers that cause the receptor to remain in a constitutively “on” state, leading to uncontrolled cell growth and survival. These mutations are most clinically significant in NSCLC and Glioblastoma (GBM), as well as in other epithelial cancers (Table 2) [150,151].

Naturally occurring mutations in the EGFR gene are most commonly found in NSCLC, especially in never-smokers, women, and individuals of East Asian descent. These mutations are primarily somatic (acquired after birth) rather than hereditary. Two mutations, deletions in exon 19 and the single amino acid substitution L858R in exon 21, which encode the tyrosine kinase domain, often referred to as “classical” EGFR mutations or common EGFR mutants. These two mutations account for approximately 85% of all EGFR mutations and approximately 85–90% of all EGFR mutations in NSCLC. Exon 19 Deletions (Ex19del) are in-frame deletions near codons 746–750 and are the most frequent, accounting for roughly half of all mutations. This category consists of over 30 different subtypes. These mutations are in-frame deletions that typically remove 3 to 7 amino acids between residues L747 and P753 in the tyrosine kinase domain (Table 3) [152].

L858R point mutation is a single amino acid substitution where leucine is replaced by arginine at codon 858 in exon 21. This mutation accounts for approximately 40–41% of EGFR mutations in NSCLC. Structural studies have shown that L858R and exon 19 deletions (Ex19Del) destabilize the inactive conformation of the receptor, leading to increased increase in dimerization and activity compared to WT. These classical mutations are highly significant because they make the cancer cells sensitive to targeted therapies called EGFR tyrosine kinase inhibitors (TKIs), such as gefitinib, erlotinib, and Osimertinib [153,154].

About 10–15% of NSCLC patients have rarer EGFR mutations, which often have different responses to standard TKI treatments. These EGFR mutants are called uncommon or atypical EGFR Mutants. The common Uncommon EGFR mutants include Exon 20 Insertions, G719X (e.g., G719S, G719A), S768I and L861Q, and T790M [155,156].

The G719X mutation in EGFR exon 18 is classified as an “uncommon” driver mutation in non-small cell lung cancer (NSCLC) that confers sensitivity to tyrosine kinase inhibitors (TKIs). Second-generation TKIs, such as afatinib, demonstrate favorable response rates, though typically not as high as those observed in common mutations. Third-generation TKIs, including osimertinib, have also been investigated, particularly in the context of complex mutations. G719X frequently co-occurs with other mutations, such as S768I and L861Q, and occasionally with E709X, which can influence TKI responsiveness. Comprehensive sequencing of exons 18–21 remains essential for optimizing therapeutic strategies [157].

As discussed above, Exon 18 and Exon 21 mutations represent two biologically and clinically distinct categories within EGFR-mutated non-small cell lung cancer (NSCLC). While Exon 21 hosts one of the highly responsive “classic” mutations, Exon 18 alterations belong to the “uncommon” family, requiring fundamentally different frontline drug selection. Second-generation irreversible covalent TKIs like afatinib are the primary treatment of choice for G719X. On the other hand, Osimertinib (80 mg daily), a third generation TKI, acts as the preferred structural blockade of L858R. It creates an irreversible covalent link inside the ATP-binding cleft to neutralize signaling [158,159,160].

Exon 20 mutations include both Exon 20 insertions and mutations such as T790M. While both are located on Exon 20 of the EGFR gene, they represent completely opposite biological mechanisms. Exon 20 insertions make up about 4–10% of all EGFR mutations and are intrinsic driver that structurally distorts the receptor, while T790M is typically an acquired gatekeeper mutation that alters chemical affinity. Exon 20 insertions add 1 to 7 amino acids directly into the loop following the regulatory C-helix. This insertion acts like a physical wedge, pushing the C-helix inward into its “always-on” active state. This movement severely shrinks and structurally compresses the drug-binding cleft. Early-generation TKIs (like erlotinib) or standard 3rd-generation TKIs (osimertinib) simply cannot physically fit inside this distorted, narrowed pocket. Because the binding cleft is narrowed, treatment requires drugs engineered with a highly compact structure or extracellular delivery. Standard treatment utilizes the bispecific antibody amivantamab combined with platinum-doublet chemotherapy to bypass the internal pocket completely by clamping down on the outside of the receptor. If the tumor progresses, specialized third generation TKIs like sunvozertinib (Zegfrovy) or furmonertinib are used. These molecules feature flexible, smaller, or uniquely angled chemical backbones capable of sliding deep inside the compressed Exon 20 insertion cleft [161,162,163].

On the other hand, For T790M, the replacement of T by M swapping a small, polar Threonine for a bulky, hydrophobic Methionine. Position 790 sits directly at the bottom of the ATP-binding pocket, acting as the “gatekeeper” residue. Methionine does not entirely block drugs physically. Instead, its massive sulfur-containing side chain drastically shifts the chemical thermodynamics of the pocket, restoring the receptor’s binding affinity for cellular ATP to normal, wild-type levels. Because 1st-generation TKIs are reversible, the high-affinity ATP molecules effortlessly kick the drug out of the pocket. Because T790M’s resistance is driven by high ATP competition, the drug must bind permanently before ATP can push it away. Treatment relies heavily on osimertinib, a third generation TKI. Osimertinib features an acrylamide group that forms an un-breakable, irreversible covalent bond with a nearby cysteine residue (Cys797) in the pocket. Once locked in, it successfully neutralizes the T790M mutant regardless of how much ATP surrounds it [164,165].

While primarily discussed in lung cancer, atypical EGFR mutations appear across several malignancies with varying clinical implications. EGFRvIII (EGFR variant III) is the most common EGFR gene mutation in glioblastoma multiforme, a highly aggressive brain cancer. Although often called an “alternative splice variant,” EGFRvIII actually results from a stable, in-frame deletion of 801 base pairs (exons 2–7) in the EGFR gene. This deletion leads the splicing machinery to connect exon 1 directly to exon 8, producing mRNA that mimics alternative splicing. Evidence indicates that this variant results from somatic architectural changes or intricate genomic rearrangements, rather than solely from regulatory errors involving the spliceosome. The associated deletion eliminates part of the extracellular domain, producing a receptor incapable of binding its standard ligands—such as EGF—yet remaining constitutively active [151,166].

In head and neck cancer mutations are rare (<5%) but include variants like EGFRvIII (found in ~20% of cases), which are linked to aggressive behavior and resistance to radiation and standard antibodies. In CRC mutations are extremely rare (<1%). It is interesting to note that EGFR eligibility for Cetuximab is actually defined by RAS/BRAF wild-type status, not by the status of the EGFR gene itself [149,167].

### 4.3. EGFR Ligand Amplification (Autocrine Signaling) and Cancers

EGFR ligand autocrine amplification is a significant mechanism of cancer progression, where tumor cells produce their own growth factors to drive uncontrolled proliferation and survival. This self-stimulation loop often occurs alongside other mechanisms, such as gene amplification and mutations, and is a key factor in tumor development, invasion, and resistance to therapies [168,169].

Cancer cells often overexpress EGFR ligands such as amphiregulin (AREG), transforming growth factor-α (TGF-α), and epiregulin (EREG). These secreted ligands bind to the EGFR on the same cell (autocrine) or neighboring cells (paracrine), leading to persistent receptor activation. Some ligands (like AREG and EREG) or cancer-associated mutations result in reduced receptor ubiquitination and degradation, causing the EGFR to be recycled back to the cell surface where it continues to signal. The enhanced ligand-dependent activation of EGFR triggers major downstream signaling pathways that promote cancer development and drug resistance. EGFR autocrine amplification mechanisms are observed in a wide range of cancers, including GBM, NSCLC, Breast Cancer, HNSCC, and Pancreatic Cancer [168,169,170].

In breast cancer, autocrine ligands like AREG and TGF-α recruit macrophages to the tumor microenvironment, which then release additional growth factors, creating a complex feedback loop that promotes angiogenesis and metastasis. In GBM, amplification of wild-type EGFR is common; however, the presence of ligands like TGF-α can actually shift the receptor’s role from promoting invasion to promoting proliferation, sometimes making the tumor less invasive but more proliferative. In pancreatic cancer: Integrin signaling cooperates with autocrine loops of AREG and Epiregulin (EREG) to drive high rates of migration and 3D invasive growth. In lung and head and neck cancers, high levels of autocrine AREG often correlate with stable disease when treated with inhibitors like gefitinib or cetuximab, serving as a potential biomarker for treatment sensitivity [168,171,172,173].

### 4.4. Impaired Downregulation/Degradation and Cancer

In healthy cells, the Epidermal Growth Factor Receptor (EGFR) is carefully regulated: after binding to its ligand (like EGF), it is internalized and sent to lysosomes for degradation, effectively “turning off” the growth signal. In many cancers, this process is broken, allowing the receptor to stay active and continuously drive cell growth.

Cancers use several mechanisms to prevent EGFR from being destroyed. These mechanisms include defective ubiquitination, hypophosphorylation of Y1045, enhanced recycling, Heterodimerization with HER2, and altered trafficking proteins.

For defective ubiquitination, the E3 ubiquitin ligase c-Cbl is the primary “tagger” that marks EGFR for destruction. Mutations in EGFR (like the common L858R mutation or exon 19 deletions) often reduce its ability to bind with c-Cbl, meaning the receptor is not tagged and escapes the lysosome. Hypophosporylation of Y1045 is another mechanism to decouple EGFR and Cbl. EGFR Y1045 is the docking point for c-Cbl. In some cancers, such as cholangiocarcinoma or those with the EGFRvIII variant, this site is not activated (phosphorylated) properly, preventing c-Cbl from attaching and triggering degradation.

Instead of being degraded, internalized EGFR could be “recycled” back to the cell surface. For example, the EGFRvIII mutant common in glioblastomas is internalized slowly and recycled efficiently, keeping it active on the membrane longer. Moreover, heterodimerization with ErbB2 (HER2) could either hinder the internalization of EGFR or enhance the recycling of EGFR. It is generally accepted that HER2 avoids efficient endocytic downregulation [174,175] which contributes to its important role in the development of various cancers [6,176]. It is further shown that EGFR endocytosis is driven by receptor dimerization and ligand-induced EGFR endocytosis requires two sets of compatible endocytic codes [89,90]. Therefore, dimerization with HER2 prevent EGFR degradation and prolong its activation and signaling [175]. Loss or downregulation of helper proteins like Sortilin or Dynamin2 can also stall the internalization process, leaving more active EGFR on the cell surface to drive tumor growth P [177,178,179].

### 4.5. EGFR Crosstalk and Cancers

EGFR crosstalk involves other receptor tyrosine kinases (RTKs) like MET, AXL, HER2, HER3, Notch, integrins, E-cadherin, and IFN-γ activating downstream effectors (RAS/RAF/MEK/ERK, PI3K/AKT/mTOR) even when EGFR is not directly stimulated, or vice versa, often leading to cancer resistance to EGFR inhibitors by activating alternative survival pathways. This cross-activation allows cancer cells to bypass targeted therapies by activating redundant or parallel pathways, promoting proliferation, invasion, and survival, making combined treatments often necessary [4,180,181,182,183].

The crosstalk between HER2 (ErbB2) and EGFR (HER1) is a fundamental driver of tumor growth and therapeutic failure in various cancers, most notably breast, lung, and colorectal cancers. As members of the same receptor tyrosine kinase family, they frequently interact through heterodimerization, creating signaling complexes that are significantly more potent than individual receptors alone [5,184]. HER2 is the “preferred partner” for other family members because it exists in a “fixed” active conformation, even without a ligand. When EGFR binds a ligand (like EGF), it often pairs with HER2 to initiate robust downstream cascades. The EGFR/HER2 heterodimer is more effective at activating survival and growth pathways than EGFR homodimers. This synergy drives the over-activation of PI3K/AKT/mTOR and RAS/RAF/MEK/ERK, promoting cell survival, protein synthesis, rapid cell proliferation and gene expression [4,5,184,185].

EGFR activation can stimulate MET, enhancing cell migration and invasion in lung cancer; conversely, MET activation can promote resistance to EGFR inhibitors, often requiring dual MET/EGFR blockade [183]. For example, MET and EGFR are promising therapeutic targets for TNBC due to their high expression in multiple molecular TNBC subtypes [186]. However, crosstalk between MET and EGFR has been implicated in therapeutic resistance to single agent use of MET or EGFR inhibitors in several cancer types. It is shown that dual inhibition of MET and EGFR is required to prevent crosstalk signaling and acquired resistance [183].

EGFR can transactivate β-catenin through the PI3K/Akt pathway, leading to increased tumor cell invasion and metastasis. Conversely, Wnt ligands can activate EGFR signaling via Frizzled receptors and metalloproteinase-mediated release of EGFR ligands [187]. Crosstalk between Wnt and EGFR signaling has been observed in various cancers. In breast cancer models, Wnt overexpression activates EGFR and MAPK, an effect prevented by EGFR inhibition or sFRP1, but not linked to TGFα induction; instead, metalloproteinase inhibitors block this activation, suggesting Wnt increases EGFR ligand availability. Wnt1 transactivates EGFR in breast cancer, and in liver models with β-catenin overexpression, EGFR is a direct Wnt target that may mediate mitogenic effects. A positive relationship also exists between EGFR mutations and nuclear β-catenin in NSCLC. Together, these studies indicate strong interplay between Wnt and EGFR pathways in cancer [188,189,190].

The crosstalk between the Notch and EGFR pathways is a critical regulatory axis in cancer, often functioning to promote tumor progression, drug resistance, and the maintenance of cancer stem cells (CSCs). Notch can compensate for EGFR inhibition by maintaining survival signals through the AKT pathway, contributing to an Epithelial–Mesenchymal Transition (EMT) phenotype. Communication between Notch and EGFR is implicated in breast, lung, and head and neck cancers [182,191,192].

### 4.6. Epigenetic Modulation

EGFR drives cancer development, progression, and treatment resistance by reshaping the cellular epigenetic landscape [193,194,195]. When EGFR is chronically activated (either by mutations like EGFRvIII or by excessive ligand stimulation), it signals directly to the cell nucleus to rewrite the transcriptional landscape by remodeling the epigenome to lock in malignancy [193,196]. Activated EGFR remodels chromatin structure at enhancer regions. In brain cancers like glioblastoma, this opens up specific chromatin paths that activate key oncogenic transcription networks, such as SOX9 and FOXG1, forcing aggressive tumor growth [193]. It is also shown that downstream EGFR signaling alters histone acetyltransferases and methyltransferases. This shifts chromatin from a closed (silent) state to an open (active) state, allowing continuous transcription of pro-survival and anti-apoptotic genes [195,197]. Cancer cells can overexpress massive quantities of the normal (wild type) EGFR protein due to epigenetic misregulation [194,198].

Epigenetic modulations also play crucial roles in driving EGFR-TKI resistance, which provides promising potential therapeutic targets and biomarkers for overcoming drug resistance [195]. For example, Targeting EHMT2 reverses EGFR-TKI resistance in NSCLC by epigenetically regulating the PTEN/AKT signaling pathway [199].

## 5. Targeting EGFR-Mediated Cell Signaling for Cancer Treatment

**Figure 4:**
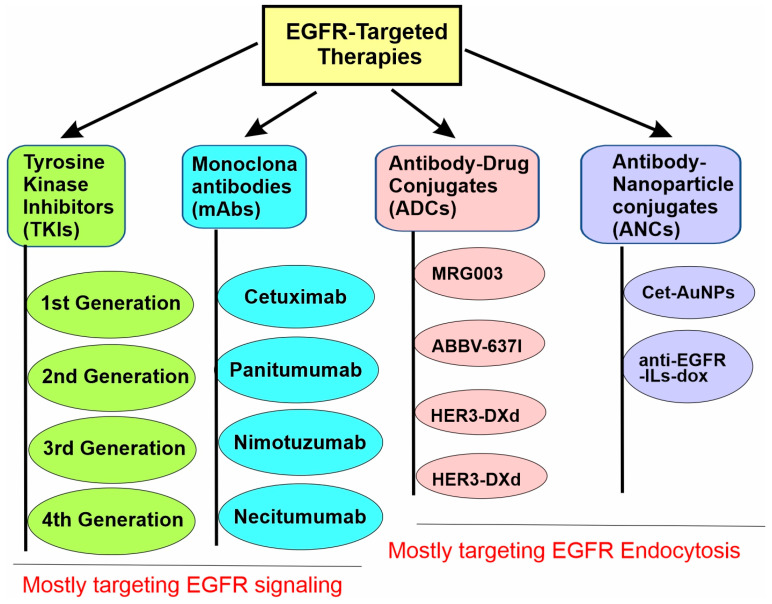
EGFR-targeted therapies.

EGFR is key in tumor development across various cancers. Mutations, overexpression, amplifications, and dysregulated EGFR signaling drive progression, especially in non–small cell lung cancer, glioblastoma, colorectal, gastric, and head and neck cancers. EGFR-targeted therapies have been developed to treat various cancers and overcome resistance. Both EGFR-mediated cell signaling and EGFR-mediated endocytosis have been targeted for cancer treatment (Figure 4).

The major strategies targeting EGFR-mediated cell signaling in cancer treatment include both monoclonal antibodies (mAbs) and tyrosine kinase inhibitors (TKI).

### 5.1. TKI

TKIs work by binding directly to the ATP-binding site within the intracellular portion of the EGFR. This prevents the receptor from sending the biochemical signals that drive cell division and tumor proliferation. Because they precisely target specific molecules involved in cancer growth, they typically cause fewer and different side effects than traditional, non-specific chemotherapy [200,201,202]. Targeting EGFR mutations with TKIs has significantly improved clinical outcomes in multiple malignancies, including lung cancer, breast cancer, CRC, and pancreatic cancer. Four generations of EGFR TKIs have been developed to suppress EGFR signaling by acting as ATP mimetics (Table 4).

The first generation of EGFR TKIs primarily includes gefitinib and erlotinib, with icotinib also recognized, particularly in China; these drugs reversibly bind to the EGFR kinase domain, blocking signals that promote cancer cell growth, and were groundbreaking in treating EGFR-mutant Non-Small Cell Lung Cancer (NSCLC). Gefitinib (Iressa), approved by FDA in 2002, is one of the earliest EGFR TKIs, approved for NSCLC patients with EGFR mutations. Erlotinib (Tarceva), approved by FDA in 2004, is another reversible inhibitor that also showed significant benefit in EGFR-mutant NSCLC. Icotinib, approved in China in 2011, shares similar mechanisms with gefitinib and erlotinib. These drugs competitively bind to the ATP-binding site within the EGFR tyrosine kinase domain. This binding prevents EGFR from activating downstream signaling pathways, halting cell proliferation and inducing cell death in cancer cells with activating EGFR mutations (such as exon 19 deletions or L858R). They revolutionized treatment for specific NSCLC patients, offering better outcomes than traditional chemotherapy for those with EGFR mutations [203].

Despite initial efficacy, resistance inevitably develops, with the T790M mutation being the most prevalent, found in approximately 50% of patients. This mutation drives resistance by enhancing ATP affinity and reducing drug binding [204,205].

To counteract T790M-mediated resistance, second-generation TKIs, such as afatinib and dacomitinib, were developed. These second-generation inhibitors irreversibly bind to EGFR at C797 and also target HER2 and HER4, broadening their therapeutic scope. They also offer improved efficacy but often with increased skin/GI toxicity and are used for EGFR-mutated NSCLC [206]. Afatinib was approved by FDA in 2013 for EGFR-mutant NSCLC and later for squamous NSCLC refractory to platinum-based chemotherapy (PBC), Dacomitinib gained approval in 2018 for metastatic NSCLC harboring Ex19del or L858R mutations [207]. These inhibitors are used in advanced NSCLC with EGFR mutations, often as first-line therapy. They provide significant response rates and improved progression-free survival (PFS) compared to chemotherapy. However, their high affinity for EGFR-WT led to dose-limiting toxicities, limiting their clinical utility. Side effects can include skin rash and diarrhea due to their broader receptor inhibition. Despite their benefits, second-generation TKIs failed to effectively overcome T790M resistance [205].

The development of third-generation EGFR TKIs addressed the limitations of prior generations by selectively inhibiting mutant EGFR while relatively sparing EGFR-WT, thereby reducing toxicity. Osimertinib (Tagrisso) are designed to target both common sensitizing EGFR mutations (like exon 19 deletions, L858R) and the T790M resistance mutation. Osimertinib offers improved control, better CNS penetration, and is now a standard first-line treatment for EGFR-mutant NSCLC. Aumolertinib is another significant third-generation TKI, particularly relevant in some regions like China. Furmonertinib, which is structurally similar to osimertinib, was approved in China in 2022 as the first-line treatment of locally advanced or metastatic NSCLC with exon 19 deletion (19DEL) or exon 21 (L858R) substitution mutations. Mobocertinib, approved in 2021, is the first EGFR TKI specifically designed for Ex20ins and has shown efficacy against rare EGFR mutations, including G719A, G719S, and S768I [208].

Despite their success, third-generation TKIs also face resistance, with the C797S mutation emerging in approximately 10% of patients, conferring resistance to osimertinib. In addition, tumors can have multiple resistance mutations (e.g., C797S + T790M), requiring drugs that can hit several targets. Fourth generation (4G) EGFR TKIs are emerging drugs designed to overcome resistance, especially the C797S mutation, as well as to target various mutant forms (including C797S alongside T790M/sensitizing mutations). They use novel mechanisms such as allosteric inhibition (binding outside the ATP pocket) for better specificity and reduced off-target effects on wild-type EGFR. The 4G EGFR TKIs have shown promise in early trials, with compounds like JIN-A02 and THE-349 in development for EGFR-mutated NSCLC [209,210].

### 5.2. Monoclonal Antibodies

Monoclonal antibodies (mAbs) targeting EGFR have been approved for cancers like colorectal and head/neck cancers. Most of these Y-shaped antibodies bind to the extracellular domain of EGFR, stopping natural growth factors (like EGF) from attaching. Prevent This binding blocks receptor dimerization and signal transduction, effectively turning off growth signals. The antibody-receptor complex is often internalized and destroyed. They also can mark cancer cells for destruction by the body’s immune system (Antibody-Dependent Cellular Cytotoxicity).

The key approved EGFR mAbs include Cetuximab, Panitumumab, Nimotuzumab, and Necitumumab. Cetuximab, approved by FDA in 2004 and sold under the brand name Erbitux, is an EGFR inhibitor used primarily for the treatment of metastatic colorectal cancer and head and neck cancer. The mechanisms underlying Cetuximab is not completely clear, but it has been shown that cetuximab blocks ligand binding to EGFR by competitively binding to specific extracellular regions of EGFR. Cetuximab also sterically hinders the binding of EGFR to other ErbB family members. In addition, it down regulates EGFR by promoting EGFR endocytosis and degradation. Moreover, cetuximab also interacts with other proteins important for cell proliferation such as p27 kip1 and proliferating cell nuclear antigen (PCNA), as well as proteins important for cell survival such as Bcl-2 (anti-apoptosis) and Bax (pro-apoptosis). Cetuximab also significantly inhibits the expression of pro-angiogenic factors, thereby reducing tumor angiogenesis [211].

Panitumumab (Vectibix) is a fully human monoclonal antibody that targets the EGFR and was approved by FDA in 2006 to treat metastatic colorectal cancer (mCRC). Panitumumab specifically binds to the extracellular domain of EGFR, inhibiting its signaling, causing cell cycle arrest, and reducing angiogenesis. It is highly effective in patients with RAS wild-type (KRAS/NRAS) metastatic colorectal cancer. Patients with mutated RAS do not benefit and may even experience harm [212].

While initially effective, patients often develop acquired resistance within 3–12 months, frequently due to the development of new mutations in the EGFR-RAS-RAF-MEK pathway. To overcome the resistance, rechallenge therapy was developed to use panitumumab as a “rechallenge” (re-introducing the drug) in combination with other treatments for patients who previously responded. In a recent clinical trial, circulating tumor DNA was used to guide rechallenge with panitumumab in metastatic colorectal cancer. This phase 2 CHRONOS trial showed that interventional liquid biopsies can be effectively and safely exploited in a timely manner to guide anti-EGFR rechallenge therapy with panitumumab in patients with mCRC [213]. Panitumumab combined with trifluridine-tipiracil improved PFS over trifluridine-tipiracil alone in patients with refractory RAS WT MCRC, supporting its clinical use. The findings support the clinical utility of liquid biopsy–guided anti-EGFR rechallenge therapy for refractory RAS WT MCRC [214].

Necitumumab is a human IgG1 monoclonal antibody that targets EGFR for treating advanced squamous non-small-cell lung cancer (NSCLC). It is approved in 2015 for first-line treatment of metastatic squamous NSCLC in combination with chemotherapy. It is not for non-squamous NSCLC. Necitumumab binds to domain III of the extracellular region of EGFR, blocking ligand binding and preventing dimerization, which leads to receptor internalization and degradation. Because it acts on the external receptor, it is being studied alongside tyrosine kinase inhibitors (TKIs) like osimertinib for overcoming resistance in EGFR-mutant cases [215].

Nimotuzumab (commercially known as CIMAher, Theracim, or BIOMAb EGFR) is a recombinant, humanized IgG1 monoclonal antibody that targets the extracellular domain of EGFR. Unlike above discussed anti-EGFR antibodies (such as cetuximab or panitumumab), nimotuzumab is famous in oncology for its “bivalent binding requirement,” which allows it to selectively destroy tumor cells while completely sparing healthy tissue [216].

## 6. Targeting EGFR Endocytosis for Cancer Drug Delivery

While EGFR-mediated cell signaling has been traditionally targeted by mAbs and TKIs to treat EGFR-expressing malignancies, EGFR-mediated endocytosis is increasingly being leveraged to deliver anticancer agents specifically into EGFR-expressing cancer cells. To achieve targeted intracellular delivery, a delivery system must possess two essential functions: recognition of the EGFR-expressing cancer cells and subsequent internalization of the therapeutic payload. Both functions can be fulfilled by anti-EGFR mAbs, such as cetuximab or its Fab fragments. Indeed, both antibody–drug conjugates (ADCs) and antibody–nanoparticle conjugates (ANCs) utilize cetuximab or other anti-EGFR mAbs to deliver therapeutic cargoes directly to EGFR-expressing cancer cells [16,17,217,218].

Cetuximab competitively binds to the extracellular domain of EGFR, ensuring the highly specific targeting of EGFR-expressing cancer cells. Beyond simple binding, cetuximab stimulates the endocytosis of the receptor, which facilitates the internalization of conjugated anticancer agents, achieving targeted inhibition of cancer cell growth and survival [219,220,221,222].

In this section, we discuss the current developments and future directions regarding how various EGFR endocytosis pathways can be hijacked to achieve efficient and specific delivery of anticancer agents into EGFR-expressing cancer cells using ADC and ANC platforms.

### 6.1. ADCs

#### 6.1.1. Overview

Antibody–drug conjugates (ADCs) are targeted cancer therapies that combine the homing precision of monoclonal antibodies (mAbs) with the cell-killing power of chemotherapy. Acting like “guided missiles,” they deliver highly potent drugs directly to tumors, thereby sparing healthy tissues and reducing the side effects associated with standard chemotherapy.

An ADC is built from three critical components: the mAb, the linker, and the payload. The mAb is engineered to specifically target tumor-cell receptors and initiate internalization of the drug via clathrin- or caveolin-mediated endocytic pathways. The linker acts as a secure chemical tether that keeps the drug attached while traveling through the bloodstream. The payload consists of a powerful chemotherapy drug or cytotoxin. Once the ADC enters the cancer cell, the linker breaks down and releases the payload to destroy the cell from within.

The endocytosis of ADCs follows several key stages. First, the antibody component of the ADC binds to a specific tumor-associated antigen on the cancer cell surface. The ADC-antigen complex is then enveloped and taken into the cell via endocytosis (predominantly clathrin-mediated). These internalized vesicles mature into early endosomes, which subsequently fuse with lysosomes. Lysosomal enzymes and the acidic environment degrade the ADC, resulting in the dissociation of the cytotoxic drug from the antibody and the subsequent release of the drug from the lysosome into the cytoplasm to exert its antitumor effect. The success of this targeted delivery relies heavily on several biological variables, including the antigen expression level, the receptor internalization rate, and lysosomal functions [223,224,225,226].

#### 6.1.2. ADCs Targeting EGFR

Antibody-drug conjugates (ADCs) targeting EGFR are an evolving therapeutic class designed to overcome resistance to standard EGFR TKIs. ADCs combine antibody tissue specificity with the high cytotoxicity of antineoplastic drugs. The antibody component is covalently attached to the drug via a chemical linker, binds to extracellular receptors, and, upon endocytosis, releases the active payload inside the cell through chemical or enzymatic cleavage to exert targeted cytotoxicity. To date, EGFR-targeted ADCs have been developed using monoclonal antibodies such as cetuximab, panitumumab, and other anti-EGFR agents paired with potent payloads like taxanes and auristatin derivatives. Current development focuses on utilizing high-potency payloads and multi-specific or bispecific designs to improve selectivity and therapeutic efficacy in malignancies such as NSCLC, colorectal cancer (CRC), and triple-negative breast cancer (TNBC).

There are at least 20 anti-EGFR ADCs concurrently moving through global preclinical and clinical pipelines. Because oncology pipelines update continuously with new trial registrations, structural designs, and licensed molecules, the absolute number of active assets fluctuates over time. However, the investigational landscape can be explicitly quantified by their progressive clinical development phases. Rakuten Medical’s Akalux^®^ (cetuximab saratolacan) represents the first EGFR-targeted conjugate approved for clinical use (specifically in Japan). Promising candidates that have reached Phase III clinical trials include CSPC Pharmaceutical’s SYS6010 (targeting EGFR-mutant non-small cell lung cancer) and BL-B01D1 (a bispecific EGFR × HER3 candidate). Historically, depatuxizumab mafodotin also reached Phase III.

Furthermore, approximately 10 anti-EGFR ADCs have progressed into Phase I/II clinical trials. Active global trials are testing monospecific molecules, such as becotatug vedotin (MRG003) and Forbius’ AVID100, alongside multi-targeting bispecific constructs, such as AZD9592 (EGFR × c-Met) and M1231 (EGFR × MUC1). The remaining pipeline candidates are currently undergoing preclinical investigation [99,227,228].

Akalux^®^ (cetuximab saratolacan sodium) is a first-in-class antibody-dye conjugate utilized in a novel oncological modality known as photoimmunotherapy. It functions by selectively targeting cancer cells and inducing rapid physical cell lysis via near-infrared light activation. Structurally, Akalux consists of two primary components covalently joined together: the cetuximab antibody backbone and the photo-absorbing dye IRDye^®^ 700DX [229].

SYS6010 is a major, rapidly advancing anti-EGFR ADC developed by CSPC Pharmaceutical Group. Unlike older anti-EGFR ADCs that rely on microtubule inhibitors like MMAE or MMAF (such as MRG003 or depatuxizumab mafodotin), SYS6010 utilizes a novel topoisomerase I inhibitor payload (designated as JS-1). This payload is attached via a cleavable tetrapeptide linker, allowing for highly potent DNA damage induction and a robust bystander-killing effect in heterogeneous tumors. Reflecting its strong clinical pipeline status, SYS6010 has moved exceptionally fast through clinical tracks into advanced phase evaluations [226,230].

Depatuxizumab mafodotin (also known as Depatux-M or ABT-414) is an early, first-generation EGFR-targeting ADC and serves as the historical predecessor to newer constructs like ABBV-637. While it showed immense preclinical promise, its clinical development was ultimately halted due to insufficient survival outcomes and severe ocular toxicities [231,232]. ABBV-637 is an investigational ADC designed to treat solid tumors, particularly EGFR-amplified glioblastoma and EGFR-mutated NSCLC. It combines an EGFR-targeting antibody with a BCL-XL inhibitor payload to induce targeted apoptosis. Although clinical trials have assessed its safety and efficacy both as a monotherapy and in combination with other agents (such as ERAS-801 or osimertinib) [233], and preliminary Phase I data was shared at late-2023 oncology congresses, AbbVie ultimately removed the drug from its active pipeline.

While Rakuten Medical’s Akalux^®^ is an antibody-dye conjugate engineered for light-activated photoimmunotherapy, Forbius’ AVID100 is a traditional ADC designed to deliver a cytotoxic payload directly into cells without physical external activation. The antibody component is an IgG1 anti-EGFR antibody engineered with the same variable region sequence as cetuximab, while the payload is DM1 (mertansine), a highly potent microtubule inhibitor. Once AVID100 binds to EGFR on the tumor cell surface, the entire receptor-drug complex is internalized. Intracellularly, the DM1 toxin is released, disrupting the cellular microtubule network, preventing mitosis, and inducing cell death [234].

MRG003 is a novel ADC consisting of an anti-EGFR humanized immunoglobulin G1 (IgG1) monoclonal antibody conjugated to the microtubule disruptor monomethyl auristatin E (MMAE) via a protease-cleavable valine-citrulline linker. The potent antitumor activity of MRG003 has been demonstrated in preclinical xenograft mouse models. Furthermore, results from a recent Phase I clinical trial suggest that MRG003 possesses an acceptable safety and tolerance profile in most patients with EGFR-expressing solid tumors, alongside encouraging therapeutic activity in patients with squamous cell carcinoma of the head and neck (SCCHN) and nasopharyngeal carcinoma (NPC) [235].

It is worth noting that both HER3-DXd (patritumab deruxtecan) and T-DXd (trastuzumab deruxtecan) are not EGFR-targeting ADCs; rather, they are used to treat malignancies that have developed resistance to standard EGFR TKI therapies. These resistant cancer cells frequently overexpress HER3 and HER2 to bypass upstream EGFR signaling blockage. Instead of fighting the primary EGFR mutation directly, HER3-DXd and T-DXd exploit these highly abundant companion receptors as alternative cellular entry pathways to internalize and deliver their respective topoisomerase I inhibitor payloads [236,237,238,239].

### 6.2. ANCs

#### 6.2.1. Overview

ADCs are powerful targeted therapies, but they face steep biological and structural limitations, including limited payload capacity, systemic toxicity from premature drug release, poor tumor penetration, and resistance mechanisms such as drug efflux pumps. ANCs overcome these weaknesses by using a nanoparticle core that carries thousands of drug molecules, isolates toxic payloads until delivery, utilizes multivalent targeting to bypass resistance, and alters release kinetics [17,18].

One of the key advantages of ANCs is their massive payload capacity. While ADCs typically carry 4–8 drug molecules per antibody, ANCs can deliver 10,000 to 1,000,000 times more drug per targeting event due to the high loading capacity of the nanoparticle core. In addition, coating a nanoparticle with multiple antibodies allows for “avidity effects,” where the conjugate binds much more strongly to target cells than a single antibody could, potentially overcoming low antigen expression. Moreover, the drug is often encapsulated non-covalently within the nanoparticle, allowing for a “plug-and-play” strategy where different drugs or antibodies can be swapped without redesigning complex chemical linkers. Finally, ANCs can be engineered to release their cargo only in specific environments (e.g., acidic tumors), reducing systemic toxicity [17].

#### 6.2.2. ANCs Targeting EGFR

Several ANCs targeting EGFR have been reported and evaluated, with some advancing through clinical trials. ANCs targeting EGFR are also referred to as EGFR-targeted immunoliposomes or antibody–functionalized nanoparticles (AFNPs) [218,240]. Various EGFR-targeting ANCs have been constructed by using different nanocarriers, including liposomal nanocontainers, polymer-based nanoparticles, polymer-lipid hybrid nanoparticles (PLHNs), gold nanoparticles, micellar nanoparticles, and magnetic nanomaterials. EGFR-targeting ANCs have been developed to treat many cancer types, including lung, head and neck, breast, brain, and colorectal cancers [241,242,243,244].

Liposomal nanocontainers represent the most clinically advanced EGFR ANCs. Anti-EGFR-ILs-dox (NCT01702129/NCT02833766) is one of the most thoroughly reported EGFR ANCs in human clinical trials. It features Fab’ fragments of the anti-EGFR monoclonal antibody cetuximab inserted into the membrane of PEGylated liposomes encapsulating doxorubicin. Following a successful Phase I trial in advanced solid tumors, a multicenter Phase II clinical trial evaluated its efficacy as a first-line treatment for patients with advanced EGFR-positive triple-negative breast cancer (TNBC) [240].

Cetuximab-DOPE/CHEMS liposomes are pH-sensitive immunoliposomes designed to deliver chemotherapy drugs, particularly docetaxel (DTX), selectively to EGFR-overexpressing cancer cells [245]. Their structure depends on two lipid components with complementary functions: DOPE (dioleoylphosphatidylethanolamine), a neutral helper lipid that favors non-lamellar inverted hexagonal phases, and CHEMS (cholesteryl hemisuccinate), an acidic cholesterol derivative that stabilizes DOPE at physiological pH. Cetuximab is covalently attached to the liposome surface, enabling specific binding to the extracellular domain of EGFR. After binding to EGFR-positive tumor cells, the liposome is internalized by endocytosis. In the acidic endosomal environment, CHEMS becomes protonated, neutralizing its negative charge and reducing electrostatic repulsion; this destabilizes the bilayer and promotes a transition to the hexagonal phase, facilitating drug release [245].

Polymer-based constructs enable precise control of drug-release kinetics and support high payload loading. Anti-EGFR PLGA-PEG nanoparticles are versatile polymeric nanocarriers designed for targeted cancer therapy. Unlike liposomes, which rely on fluid lipid bilayers, these nanoparticles contain a solid, biocompatible polymeric core that supports the stable, controlled release of hydrophobic drugs. By combining the structural stability of PLGA-PEG with the targeting capability of anti-EGFR antibodies such as cetuximab, this platform can deliver therapeutic payloads directly to EGFR-overexpressing tumors, including triple-negative breast cancer, glioblastoma, lung cancer, and colorectal cancer [246,247,248,249]. In a recent study, a polymeric nanoparticle fabricated with anti-EGFR mAb and 5-fluorouracil (5-FU) (anti-EGFR-5-FU-PLGA-PEG-NP) was developed to target EGFR-expressing colorectal cancer. This EGFR-targeting ANC shows biphasic drug release kinetics, higher cellular uptake, and higher cytotoxicity, holding excellent potential for drug delivery to EGFR-positive colorectal cancer cells [250]. This technology has also been used to construct anti-EGFR-SPIONs as targeted magnetic resonance imaging (MRI) contrast agents for the EGFR-specific detection of tumor cells [251].

Unlike a polymer nanoparticle, which consists of a uniform, solid plastic matrix, a hybrid nanoparticle wraps that solid polymer core in a fluid, biological lipid membrane. This hybrid layout directly mimics the outer shell of a natural virus or cell to achieve better stability in human blood. Polymer-Lipid Hybrid Nanoparticles (PLHNs) consist of a solid polymer center (typically PLGA) that provides structural mechanical strength and holds a high payload of hydrophobic drugs, a layer of neutral lipids (such as lecithin or phosphatidylcholine) that binds directly to the polymer core, and a layer of PEGylated lipids (such as DSPE-PEG) that extends outward. This outer layer prevents blood proteins from sticking to the nanoparticle, keeping it hidden from the immune system. Compared with pure polymer nanoparticles, the hybrid offers superior release control, high structural stability, high payload flexibility, and easier surface customization [249].

Hybrid nanoparticles have been widely employed to target EGFR. In an early study, PLNP-Mal-EGFR was created and showed significantly enhanced cellular cytotoxicity against hepatocellular carcinoma (HCC) cells overexpressing EGFR. PLNP-Mal-EGFR uses polymer-lipid hybrid nanoparticles containing DSPE-mPEG as a nanocarrier and the cetuximab Fab fragment as a targeting antibody [252]. In a more recent study, EGFR-targeted and NIR-responsive hybrid lipid-polymer nanoparticles (Cet-CINPs) were prepared for the targeted delivery of irinotecan. Cet-CINPs showed pH/NIR-triggered drug release behavior and achieved effective chemo-photothermal therapy against LoVo cells. High cytotoxicity of Cet-CINPs was achieved owing to the photothermal effect of ICG when irradiated by an NIR laser [253].

EGFR-targeted thermosensitive liposomes (TSLs) are advanced, dual-mechanism nanocarriers engineered for cancer therapy to achieve highly precise, localized drug delivery. This dual-targeting framework combines active biological targeting (via EGFR antibodies or peptides) with physical stimuli-responsive targeting (via heat-triggered cargo release) to maximize cytotoxicity at the tumor site while minimizing systemic side effects. In an early study, TSLs were combined with an EGFR-specific peptide (GE11) and Fab’ fragments of cetuximab. The Fab’-decorated TSLs (Fab’-TSLs) bound to EGFR-overexpressing cancer cells more specifically and efficiently than the GE11-modified TSLs. Fab’-conjugation combined with hyperthermia induced enhanced tumor cell cytotoxicity of doxorubicin-loaded TSLs, showing great potential for combinational targeted and triggered-release drug delivery [254]. More recently, the magnetic potential of iron oxide was explored within the TSL system. Citric acid-coated iron oxide magnetic nanoparticles (CMNPs) were encapsulated into thermo-sensitive liposomes (TSLs). The chemotherapeutic drug doxorubicin (DOX) was then loaded into the CMNP-TSLs, which were coated with cetuximab to target EGFR-expressing breast cancer cells in in vitro and in vivo mouse models. The study revealed that a combined approach of photothermal therapy and targeted chemotherapy using thermo-sensitive nanocarriers represents a promising therapeutic strategy against breast cancer [255].

Cetuximab-conjugated gold nanoparticles (Cet-AuNPs) are multifunctional nanostructures used in cancer research for targeted drug delivery, imaging, and thermal therapy. By attaching the monoclonal antibody cetuximab to a gold nanoparticle core, researchers can deliver concentrated therapeutic effects directly to tumor cells overexpressing EGFR [256]. Liposomes—spherical vesicles composed of phospholipids and cholesterol that can encase both lipophilic and hydrophilic drugs—are another widely studied category of nanoparticle. These structures can be functionalized with anti-EGFR antibodies through techniques such as maleimide modification to produce immunoliposomes, which have shown strong in vitro and in vivo results. Currently, an anti-EGFR immunoliposome encapsulating doxorubicin (anti-EGFR-ILs-dox) is undergoing Phase II clinical trials for triple-negative, EGFR-positive breast cancer and Phase I trials for high-grade gliomas, according to FDA clinical trial data [99].

Anti-EGFR lipid micellar nanoparticles are advanced, self-assembling nanostructures designed for targeted cancer therapy, specifically optimized to penetrate dense tumor tissues and selectively deliver therapeutic payloads to EGFR-overexpressing malignancies. These platforms achieve significantly higher accumulation inside tumor cells compared to traditional, larger liposomes. Researchers have developed anti-EGFR lipid micellar nanoparticles that co-encapsulate quantum dots and paclitaxel for targeted tumor imaging and therapy. In vivo testing showed that antibody– or aptamer-conjugated micelles, particularly in EGFR-positive tumor models, enhanced delivery and significantly reduced tumor growth compared to non-targeted formulations [243]. In a recent study, EGFR-targeted, pH-sensitive micelles-in-lipopolymersome nanocarriers were developed to overcome multidrug resistance in triple-negative breast cancer. This hybrid system co-delivers hydrophobic and hydrophilic drugs, demonstrating superior tumor inhibition and reduced toxicity in animal models [257].

Despite their potential, ANCs face significant obstacles in clinical translation, including the formation of a protein corona in the bloodstream, potential immunogenicity, and the need for standardized, scalable, and reproducible synthesis methods. Future developments must focus on optimizing antibody density and orientation, as well as improving overall tumor specificity to reduce off-target effects [258].

### 6.3. Exploring Various EGFR Endocytosis to Improve ADC/ANC Delivery

While drug delivery efficiency can be improved by modifying the nanoparticles used in ANCs, further exploration of EGFR endocytosis pathways remains a highly promising strategy to enhance the delivery of both ADCs and ANCs. As discussed above, EGFR endocytosis occurs through several distinct routes, including ligand-induced canonical pathways (such as CME and NCE) and the non-ligand-induced, non-canonical, p38-mediated pathway (which occurs via CME). These endocytosis pathways are currently being explored to improve the delivery efficiency of ADCs and ANCs.

Most ADCs and ANCs employ either the full cetuximab antibody or the Fab fragment of cetuximab to target EGFR. While the mechanisms of EGFR endocytosis initiated by cetuximab binding are not fully understood, it is generally accepted that internalization occurs via CME, albeit at a much slower rate than ligand-induced EGFR endocytosis [221,222]. Therefore, enhancing cetuximab-induced EGFR endocytosis represents a potential strategy to improve drug delivery efficiency.

#### 6.3.1. Targeting Non-Ligand-Induced, p38-Mediated EGFR Endocytosis for Drug Delivery

Targeting the p38-mediated endocytosis of EGFR is an emerging, cutting-edge strategy to significantly boost the intracellular delivery of ADCs and ANCs. Because standard EGFR-targeting drugs (like cetuximab) often show poor internalization, researchers are utilizing the p38 stress-induced kinase pathway. Activating this pathway—either chemically (e.g., using TNF-α or chemotherapy agents like cisplatin) or via physical stress—stimulates non-canonical EGFR endocytosis. This process rapidly pulls drug-receptor complexes into the cell [100,242,259]. Consequently, this strategy offers enhanced internalization and helps overcome drug resistance. ANCs could therefore be designed to target EGFR while simultaneously modulating the ROS-p38 axis to induce synergistic cancer cell apoptosis [260].

Following its identification, the p38-mediated endocytosis pathway was immediately explored for cancer therapy. Research demonstrated that p38-mediated endocytosis enhances the cytotoxic effect of drugs like cisplatin by preventing the survival signaling normally induced by internalized EGFR [93]. It was further shown that cisplatin induces cellular stress in non-small cell lung cancer (NSCLC) cells, activating the p38 MAPK pathway to trigger the non-canonical, monomeric endocytosis of EGFR. The mechanism involves p38-mediated phosphorylation of EGFR at the C-terminal Ser-1015 residue, which facilitates receptor internalization independently of ligand binding or tyrosine kinase dimerization [37].

Temozolomide (TMZ) is a chemotherapy drug that typically functions as a DNA alkylating agent. Interestingly, beyond this standard role, TMZ induces cellular stress that triggers the endocytic trafficking (internalization) of serine-phosphorylated EGFRvIII. This internalization occurs via the p38-mediated non-canonical pathway. When researchers introduced the selective p38 inhibitor SB203580, both the serine phosphorylation and the internalization of EGFRvIII were completely blocked. Conversely, the tyrosine kinase inhibitor gefitinib did not halt this internalization, proving that the mechanism is driven by stress-induced p38 rather than standard EGFR kinase activation [261].

More recently, Takahashi et al. focused on ligand-independent, non-canonical, p38-mediated EGFR endocytosis to deliver ADCs into cells. They demonstrated that TNF-α induces rapid endocytosis of the cetuximab-EGFR complex within 15 min via p38 phosphorylation. This proved that ligand-independent, non-canonical endocytosis can synchronously deliver ribosome-inactivating payloads (such as saporin) into cancer cells. The study also identified that chemotherapies like cisplatin and temozolomide naturally trigger this p38 internalization mechanism [100]. In a follow-up study, this model was expanded to PC-9 non-small cell lung cancer (NSCLC) cells harboring EGFR exon 19 deletions. By using TNF-α or cisplatin to drive p38-dependent phosphorylation, researchers achieved a stark increase in the internal uptake and cytotoxicity of a cetuximab-MMAE (monomethyl auristatin E) ADC [259].

#### 6.3.2. Utilizing Other Endocytosis Pathway

Gefitinib-Mediated Endocytosis (GME) is a phenomenon where the tyrosine kinase inhibitor (TKI) gefitinib paradoxically triggers a massive, non-physiological internalization of EGFR into intracellular compartments. While clathrin-mediated endocytosis normally acts as a pathway to degrade receptors and turn off oncogenic signaling, gefitinib-induced endocytosis acts as a double-edged sword that can either dictate drug resistance or provide new therapeutic opportunities [262,263].

When gefitinib binds to the ATP-binding pocket of EGFR, it blocks kinase activity but induces structural shifts that accelerate receptor internalization [262,263,264]. This drug-induced trafficking process relies heavily on key endocytic proteins, including Dynamin 2 (DNM2), the small GTPase Rab5, and LDL receptor-related protein 1 (LRP-1) [262]. Studies reveal that gefitinib forces EGFR to undergo joint endocytosis with other critical surface proteins, most notably the fibronectin receptor α5β1 integrin, causing them to pool closely together inside early endosomes [264].

In gefitinib-resistant cells expressing either wild-type or mutant EGFR, GME causes the internalized EGFR to aggregate in early endosomes rather than traveling to lysosomes for permanent destruction (downregulation) [265]. While trapped inside these endosomes, or rapidly recycled back to the plasma membrane by companion proteins like Rab25 or DNAJC5, the receptor continues to fire pro-survival and proliferative signals. This allows cancer cells to bypass the apoptotic effects of the drug. However, CME inhibition combined with gefitinib treatment has been shown to decrease cell survival and induce apoptosis in gefitinib-refractory cell lines, including both glioblastoma cells and wild-type EGFR NSCLC cells [262,265,266].

On the positive side, GME offers an excellent opportunity to enhance drug delivery. Because gefitinib triggers a high volume of receptor internalization, combining it with next-generation therapeutics causes cancer cells to rapidly swallow therapeutic payloads. Co-treatment with gefitinib profoundly strengthens the cellular uptake and overall toxicity of ADCs and therapeutic aptamer-siRNA complexes, effectively turning a resistance mechanism into an efficient delivery vehicle [263].

GME should be further explored in combination with ANCs. This approach does not rely on gefitinib to kill cancer cells, but rather leverages its ability to induce GME, allowing ANCs to carry massive and multiple anticancer agents into the cell.

#### 6.3.3. Antibody Engineering to Improve Drug Delivery

Antibody engineering improves EGFR-targeted drug delivery by overcoming the “binding site barrier,” bypassing drug resistance, and eliminating off-target toxicities such as severe skin rashes. Because wild-type EGFR is heavily expressed on healthy skin and gut epithelial cells, standard antibodies often cause high systemic toxicity. Engineered antibody formats act as highly specialized homing mechanisms to deliver cytotoxic drugs, nucleic acids, or nanoparticles selectively to tumor cells [267,268,269].

One approach is the generation of bispecific and multispecific antibodies [270]. Instead of targeting EGFR alone, engineered bispecific antibodies bind to two different antigens simultaneously, significantly increasing tumor selectivity and drug delivery precision. For example, molecules engineered to target both EGFR and c-Met (such as amivantamab) or EGFR and HER3 prevent cancer cells from switching to alternative survival pathways when EGFR is blocked [271,272]. Another strategy is to engage T-cells or NK-cells alongside EGFR targeting. In this setup, one arm of the antibody binds to EGFR on the tumor cell, while the other arm binds to CD3 on T-cells or CD16 on NK-cells. This physically recruits the patient’s immune cells directly to the tumor site to deliver cytotoxic granzymes and perforins [273,274,275].

In addition, prodrug antibody technology is specifically designed to eliminate the severe skin and gastrointestinal toxicities associated with wild-type EGFR targeting. In this approach, the EGFR-binding region of the antibody is chemically masked using a synthetic peptide linker, preventing it from binding to healthy tissues. When the masked antibody enters the tumor microenvironment, tumor-secreted proteases (such as matrix metalloproteinases) cleave the peptide linker. This unmasks the antibody, allowing it to bind to EGFR and deliver its therapeutic payload strictly within the tumor [276].

Another approach relies on fragment-based delivery. Full-sized monoclonal antibodies (~150 kDa) often suffer from poor tumor penetration due to the “binding site barrier,” where they bind tightly to the tumor periphery but fail to reach the core. Engineered fragments, such as single-chain variable fragments (scFv, ~25 kDa) or VHH nanobodies (~15 kDa), are significantly smaller, allowing them to penetrate deep into solid tumors and distribute drugs evenly. These small fragments are frequently engineered onto the surface of liposomes, polymeric nanoparticles, or exosome drug carriers. They act as precise, low-immunogenic targeting systems that drive ANCs to undergo EGFR endocytosis without triggering a massive immune clearance response [277,278].

## 7. Future Perspectives

Research into targeting EGFR for cancer treatment is currently focused on overcoming drug resistance, refining patient selection through precision biomarkers, and developing next-generation therapeutic modalities. The emerging trend is shifting from broad inhibition to high-precision molecular strategies designed to overcome the “inevitable” drug resistance seen with current treatments [201].

### 7.1. TKIs

Resistance remains the primary hurdle in EGFR-targeted therapy, with research evolving across distinct generations of inhibitors. Beyond the continuous development of third-generation TKIs, researchers are developing entirely new classes of drugs that go beyond standard small-molecule inhibitors. These fourth-generation TKIs, which include newer agents such as JIN-A02, are designed to target the C797S mutation, which frequently emerges as a resistance mechanism to the third-generation drug osimertinib [209].

### 7.2. mAbs

The future of anti-EGFR mAbs centers on overcoming drug resistance and improving response durability. The field is shifting toward multi-specific targeting and biomarker-driven combinations to bypass compensatory signaling pathways and directly eradicate cancer cells. Key advancements and research directions shaping the future of EGFR mAbs include bispecific antibodies, such as amivantamab, which target multiple receptors (e.g., EGFR and c-Met or HER3) simultaneously. Another key direction is dual EGFR blockade, which combines mAbs (such as cetuximab) with intracellular TKIs to create a “sandwich” effect. This intensive vertical inhibition has shown promise in delaying resistance in non-small cell lung cancer (NSCLC) and metastatic colorectal cancer (mCRC) [211,218].

### 7.3. ADCs

Antibody––drug conjugates (ADCs) targeting the EGFR pathway have reached pivotal milestones, shifting from historical design failures to clinical approvals and next-generation multi-specific platforms. The future of direct EGFR targeting relies heavily on avoiding healthy tissue uptake. Consequently, researchers are focusing on biparatopic antibodies (which target two separate regions of the EGFR protein to boost tumor internalization) and extracellularly cleavable linkers that rely on the tumor microenvironment to trigger payload release, thereby avoiding toxicities in the skin and eyes.

One major strategy is the generation of bispecific ADCs (e.g., MRG008) that combine EGFR with a second solid tumor antigen (such as 5T4). This type of ADC minimizes off-tumor toxicities by requiring dual-antigen binding. Another strategy involves dual-payload formats, which conjugate two distinct cytotoxic drugs (e.g., MMAE combined with a topoisomerase inhibitor). These ADCs can eradicate highly heterogeneous tumor clones and prevent single-agent resistance. Finally, exploring immune system synergy by combining EGFR ADCs with programmed death-1 (PD-1) inhibitors represents a promising direction. These combinations induce immunogenic cell death, turning “cold” tumors “hot” to maximize overall survival [279,280,281,282].

### 7.4. ANCs

ANCs targeting the epidermal growth factor receptor (EGFR) represent the next clinical evolution beyond conventional ADCs. By pairing the strict cellular precision of anti-EGFR monoclonal antibodies (mAbs) with the massive loading capacity of multifunctional nanomaterials, ANCs overcome the structural payload limitations inherent to ADCs. While early nanoformulations faced translational bottlenecks, such as rapid hepatic clearance and protein corona interference, emerging frameworks are shifting ANCs toward multivalent, biomarker-agnostic, and smart-responsive drug delivery platforms [16,218].

The immediate priority for future ANC commercialization is resolving the “protein corona phenomenon.” When injected into the bloodstream, nanoparticles are instantly coated by plasma proteins, masking the anti-EGFR antibodies and triggering premature immune clearance. Future designs focus on utilizing stealth zwitterionic polymers and pre-cloaking nanoparticles with patient-derived cell membranes to preserve the antibody’s target recognition until it arrives at the tumor site [258].

Another future direction is the creation of stimuli-responsive “smart” release systems. The next wave of EGFR ANCs will rely on microenvironment-specific triggers. Instead of allowing passive leaking, these smart nanoparticles remain completely inert in healthy tissue. Once they reach the tumor site, the extracellular matrix triggers drug release via localized physiological cues [258].

The ultimate goal for EGFR ANCs is the unification of diagnostics and treatment into a single theranostic workflow. Integrating reporter molecules (such as quantum dots, near-infrared fluorescence dyes, or PET/CT radioisotopes) directly into the core of an EGFR-targeted gold or iron-oxide nanoparticle will enable clinicians to non-invasively map out a patient’s intratumoral heterogeneity. This strategy allows them to visualize real-time drug delivery while simultaneously activating photothermal or radiofrequency ablation to destroy the tumor [279,283].

### 7.5. Exploring Non-Canonic EGFR Endocytosis Pathways

#### 7.5.1. p38 Mediated Endocytosis

Exploring p38-mediated endocytosis represents a paradigm shift in drug delivery. It transitions targeting strategies from relying purely on passive ligand-receptor binding to actively hijacking a cell’s internal stress response mechanisms.

A primary limitation of traditional ADCs is that certain monoclonal antibodies (such as cetuximab) block ligand binding but fail to induce robust receptor internalization, stranding the payload on the cell surface. Future therapies will utilize p38-primed combination regimens. Administering low-dose, non-lethal stressors (such as cisplatin, temozolomide, or TNF-α) strongly activates p38. This causes rapid, synchronous internalization of stalled surface antibody–receptor complexes, flooding the cell with the therapeutic payload within 15 min [100].

Instead of relying on an external drug to activate p38, next-generation nanoparticles could be engineered to carry a surface-bound or rapidly leaking stress-inducing element. Upon making initial contact with the tumor cell, the nanoparticle generates localized oxidative stress (via trace reactive oxygen species generation or localized photothermal heating). This local stress forces the immediate, self-induced activation of the host cell’s p38 pathway, essentially prompting the target cell to rapidly swallow the nanoparticle [284,285].

Cancer cells frequently resist small-molecule chemotherapies by upregulating P-glycoprotein (P-gp) and other ATP-binding cassette (ABC) efflux transporters on their plasma membrane [286]. Because endocytosis naturally pulls patches of the plasma membrane inward to form vesicles, another future strategy is to stimulate hyper-active p38-mediated endocytosis. This process rapidly internalizes these efflux pumps away from the cell surface and targets them for lysosomal degradation, simultaneously forcing drug uptake while destroying the cell’s physical defense mechanism.

Overcoming the blood–brain barrier (BBB) is critical for treating brain metastases. Because the p38 pathway modulates the structural sorting behavior of the GDI:Rab5 complex, future neurological delivery systems could utilize p38-activating transient peptides. This can temporarily shift endothelial vesicle trafficking away from lysosomal destruction and redirect it toward the transcytotic pathway, cleanly depositing neurotherapeutics into the brain parenchyma [287,288,289].

#### 7.5.2. GME

Utilizing GME in combination with ADCs and ANCs represents an innovative mechanism for EGFR-targeted oncological drug delivery. The future of GME lies in shifting its clinical use away from monotherapy and repurposing it as a highly active, synchronous endocytic priming engine to force the uptake of EGFR-directed conjugates and nanomedicines. Gefitinib could be used as a sequential priming agent to pull surface-bound ADCs rapidly into the early endosome network. This strategy directly resolves the clinical bottleneck of insufficient antibody internalization. Recent preclinical benchmarks indicate that GME significantly potentiates the efficacy of aptamer-siRNA conjugates. Consequently, future therapeutic delivery platforms could leverage GME to safely deliver gene-silencing therapeutics into aggressive, overexpressing epithelial tumors. Furthermore, GME could be combined with payload delivery to simultaneously strip away a glioma cell’s capacity to invade healthy brain tissue while pulling therapeutic nanocarriers across the cell membrane [262,263,264,290].

#### 7.5.3. Others

Proteolysis Targeting Chimeras (PROTACs), another type of fourth-generation TKI, use the cell’s own waste-disposal system (the ubiquitin-proteasome pathway) to completely degrade the EGFR protein rather than just blocking it. Unlike traditional inhibitors, PROTACs can be “recycled” by the cell to destroy multiple target proteins. Similarly to PROTACs, Lysosome-Targeting Chimeras (LYTACs) target extracellular and membrane-bound EGFR for destruction via the lysosome, offering a way to eliminate proteins that were previously considered “undruggable” [201].

### 7.6. Combination Therapies

Because cancers often bypass EGFR inhibition by activating alternative signaling pathways, combination therapies have shown promising results and could be another major focus for future development. Potential combination regimens include dual-receptor targeting, downstream pathway blockades, and immune system integration. Dual targeting focuses on the simultaneous inhibition of EGFR and other receptors, such as c-Met, HER2, or HER3. For example, combining amivantamab (an EGFR/c-Met bispecific antibody) with lazertinib has shown promising systemic and intracranial disease control. Researchers are also testing combinations that target the intracellular signaling cascades activated by EGFR, such as the PI3K/Akt/mTOR and MAPK/ERK pathways, to prevent tumor cells from surviving despite upstream EGFR inhibition. Furthermore, future trials are exploring the use of therapeutic cancer vaccines (e.g., targeting the EGFRvIII mutation in glioblastoma) and combining EGFR inhibitors with immune checkpoint inhibitors, such as PD-1/PD-L1 blockers, although this approach remains a complex area due to potential overlapping toxicities [291].

## Figures and Tables

**Table 1:** Clinical Impact by Cancer Type.

Cancer Type	Frequency of Gene Amplification	Clinical Significance
Head and Neck (HNSCC)	80–100%	Correlated with poor survival and radiation resistance.
NSCLC	40–80%	Strongest predictor of response to targeted Tyrosine Kinase Inhibitors (TKIs).
Glioblastoma	>50%	Often involves the EGFRvIII variant; associated with aggressive behavior.
Breast (TNBC/IBC)	30–50%	Indicator of poor prognosis; often found in Triple-Negative and Inflammatory subtypes.
Colorectal (CRC)	1–2%	positive predictive biomarker for response to anti-EGFR therapies.

**Table 2:** Common Activating Mutations by Cancer Type.

Cancer Type	Primary Activating Mutations	Mutation Location	Clinical Sensitivity
NSCLC	Exon 19 Deletions (~45%) and L858R Point Mutation (~40%)	Intracellular (Kinase Domain)	Highly sensitive to standard EGFR TKIs (e.g., Osimertinib).
Glioblastoma	EGFRvIII (~50% of amplified cases)	Extracellular (Ligand-binding domain)	Generally resistant to first/second- generation TKIs; emerging data for third-generation TKIs.
Other Cancers	Rare point mutations (G719X, L861Q)	Kinase Domain	Variable; often require second-generation TKIs like Afatinib.

**Table 3:** Most Common Sequence Variants of EGFR Exon 19 deletions.

Mutation Subtype	Frequency	Altered Sequence (Protein Level)
E746_A750del	~63–69%	Deletes ELREA string. The sequence jumps from K745 directly to T751.
L747_P753delinsS	~6–8%	Deletes LREATSP and replaces them with a single Serine (S).
L747_T751del	~4–5%	Deletes LREAT. The sequence jumps from E746 to S752.
L747_A750delinsP	~3–4%	Deletes LREA and replaces them with a Proline (P).
E746_S752delinsV	~3–4%	Deletes ELREATS and replaces them with a Valine (V).

**Table 4:** Comparison of EGFR-TKI Generations.

Generation	Representative Drugs	Mechanism of Action	Primary Target/Indication	Common Resistance Mechanism
First	Gefitinib, Erlotinib	Reversible ATP-competitive binding	Common mutations (Exon 19 del, L858R)	T790M mutation (50% of cases)
Second	Afatinib, Dacomitinib	Irreversible covalent binding to ErbB family	Common and some uncommon mutations	T790M mutation
Third	Osimertinib	Irreversible; spares wild-type EGFR	T790M-positive and first-line common mutations	C797S mutation
Fourth	BDTX-1535, BI4020	ATP-competitive, allosteric, or ortho-allosteric	C797S-mediated resistance; triple mutants	Currently being studied in clinical trials

## Data Availability

No new data were created or analyzed in this study. Data sharing is not applicable to this article.

## References

[1] Ushiro H., Cohen S. (1980). Identification of phosphotyrosine as a product of epidermal growth factor-activated protein kinase in A-431 cell membranes. J. Biol. Chem..

[2] Cohen P. (2002). The origins of protein phosphorylation. Nat. Cell Biol..

[3] Lemmon M.A., Schlessinger J., Ferguson K.M. (2014). The EGFR family: Not so prototypical receptor tyrosine kinases. Cold Spring Harb. Perspect. Biol..

[4] Wee P., Wang Z. (2017). Epidermal Growth Factor Receptor Cell Proliferation Signaling Pathways. Cancers.

[5] Wang Z. (2017). ErbB Receptors and Cancer. ErbB Receptor Signaling.

[6] Yarden Y., Sliwkowski M.X. (2001). Untangling the ErbB signalling network. Nat. Rev. Mol. Cell. Biol..

[7] Yarden Y., Pines G. (2012). The ERBB network: At last, cancer therapy meets systems biology. Nat. Rev. Cancer.

[8] Lemmon M.A., Schlessinger J. (2010). Cell signaling by receptor tyrosine kinases. Cell.

[9] Hynes N.E., Lane H.A. (2005). ERBB receptors and cancer: The complexity of targeted inhibitors. Nat. Rev. Cancer.

[10] Rexer B.N., Chanthaphaychith S., Dahlman K., Arteaga C.L. (2014). Direct inhibition of PI3K in combination with dual HER2 inhibitors is required for optimal antitumor activity in HER2+ breast cancer cells. Breast Cancer Res..

[11] Downward J., Yarden Y., Mayes E., Scrace G., Totty N., Stockwell P., Ullrich A., Schlessinger J., Waterfield M.D. (1984). Close similarity of epidermal growth factor receptor and v-erb-B oncogene protein sequences. Nature.

[12] Liu H., Zhang B., Sun Z. (2020). Spectrum of EGFR aberrations and potential clinical implications: Insights from integrative pan-cancer analysis. Cancer Commun..

[13] Liu S., Li M., Liu Y., Geng R., Ji J., Zhang R. (2025). Pan-cancer Comprehensive Analysis Identified EGFR as a Potential Biomarker for Multiple Tumor Types. Appl. Biochem. Biotechnol..

[14] Yamaoka T., Ohba M., Ohmori T. (2017). Molecular-Targeted Therapies for Epidermal Growth Factor Receptor and Its Resistance Mechanisms. Int. J. Mol. Sci..

[15] Sha C., Lee P.C. (2024). EGFR-Targeted Therapies: A Literature Review. J. Clin. Med..

[16] Md Moidul I., Harmanjot K., Harpreet K., Sushil Kumar S., Jyotibikash K., Amit K., Akashdeep S. (2025). Nanotechnology in Anti-EGFR Treatments: Enhancing Delivery and Minimizing Toxicity in Cancer Therapy. Clin. Cancer Drugs.

[17] Adhikari A., Chen I.A. (2025). Antibody-Nanoparticle Conjugates in Therapy: Combining the Best of Two Worlds. Small.

[18] Zhou X., Han Y., Fang Y., Ma P., Zhou J., Jiang Y., Xing S., Tang Q., Hou Y., Wang S. (2025). Antibody-drug conjugates: Current challenges and innovative solutions for precision cancer therapy. Med.

[19] Ullrich A., Coussens L., Hayflick J.S., Dull T.J., Gray A., Tam A.W., Lee J., Yarden Y., Libermann T.A., Schlessinger J. (1984). Human epidermal growth factor receptor cDNA sequence and aberrant expression of the amplified gene in A431 epidermoid carcinoma cells. Nature.

[20] Kovacs E., Zorn J.A., Huang Y., Barros T., Kuriyan J. (2015). A structural perspective on the regulation of the epidermal growth factor receptor. Annu. Rev. Biochem..

[21] Carpenter G., Cohen S. (1990). Epidermal growth factor. J. Biol. Chem..

[22] Cymer F., Schneider D. (2010). Transmembrane helix-helix interactions involved in ErbB receptor signaling. Cell Adh Migr..

[23] Stamos J., Sliwkowski M.X., Eigenbrot C. (2002). Structure of the epidermal growth factor receptor kinase domain alone and in complex with a 4-anilinoquinazoline inhibitor. J. Biol. Chem..

[24] Zhang X., Gureasko J., Shen K., Cole P.A., Kuriyan J. (2006). An allosteric mechanism for activation of the kinase domain of epidermal growth factor receptor. Cell.

[25] Chia C.M., Winston R.M., Handyside A.H. (1995). EGF, TGF-alpha and EGFR expression in human preimplantation embryos. Development.

[26] Normanno N., De Luca A., Bianco C., Strizzi L., Mancino M., Maiello M.R., Carotenuto A., De Feo G., Caponigro F., Salomon D.S. (2006). Epidermal growth factor receptor (EGFR) signaling in cancer. Gene.

[27] Singh B., Carpenter G., Coffey R.J. (2016). EGF receptor ligands: Recent advances. F1000Research.

[28] Batzer A.G., Rotin D., Urena J.M., Skolnik E.Y., Schlessinger J. (1994). Hierarchy of binding sites for Grb2 and Shc on the epidermal growth factor receptor. Mol. Cell. Biol..

[29] Zhang Y., Wolf-Yadlin A., Ross P.L., Pappin D.J., Rush J., Lauffenburger D.A., White F.M. (2005). Time-resolved mass spectrometry of tyrosine phosphorylation sites in the epidermal growth factor receptor signaling network reveals dynamic modules. Mol. Cell. Proteom..

[30] Roskoski R. (2014). The ErbB/HER family of protein-tyrosine kinases and cancer. Pharmacol. Res..

[31] Brown W.J., DeWald D.B., Emr S.D., Plutner H., Balch W.E. (1995). Role for phosphatidylinositol 3-kinase in the sorting and transport of newly synthesized lysosomal enzymes in mammalian cells. J. Cell Biol..

[32] Lemmon M.A., Pinchasi D., Zhou M., Lax I., Schlessinger J. (1997). Kit receptor dimerization is driven by bivalent binding of stem cell factor. J. Biol. Chem..

[33] Liu P., Cleveland T.E.T., Bouyain S., Byrne P.O., Longo P.A., Leahy D.J. (2012). A single ligand is sufficient to activate EGFR dimers. Proc. Natl. Acad. Sci. USA.

[34] Sato K. (2013). Cellular functions regulated by phosphorylation of EGFR on Tyr845. Int. J. Mol. Sci..

[35] Beguinot L., Hanover J.A., Ito S., Richert N.D., Willingham M.C., Pastan I. (1985). Phorbol esters induce transient internalization without degradation of unoccupied epidermal growth factor receptors. Proc. Natl. Acad. Sci. USA.

[36] Heisermann G.J., Wiley H.S., Walsh B.J., Ingraham H.A., Fiol C.J., Gill G.N. (1990). Mutational removal of the Thr669 and Ser671 phosphorylation sites alters substrate specificity and ligand-induced internalization of the epidermal growth factor receptor. J. Biol. Chem..

[37] Tanaka T., Zhou Y., Ozawa T., Okizono R., Banba A., Yamamura T., Oga E., Muraguchi A., Sakurai H. (2018). Ligand-activated epidermal growth factor receptor (EGFR) signaling governs endocytic trafficking of unliganded receptor monomers by non-canonical phosphorylation. J. Biol. Chem..

[38] Ullah R., Yin Q., Snell A.H., Wan L. (2022). RAF-MEK-ERK pathway in cancer evolution and treatment. Semin. Cancer Biol..

[39] Downward J. (1998). Mechanisms and consequences of activation of protein kinase B/Akt. Curr. Opin. Cell Biol..

[40] Castellano E., Downward J. (2011). RAS Interaction with PI3K: More Than Just Another Effector Pathway. Genes Cancer.

[41] Hanada M., Feng J., Hemmings B.A. (2004). Structure, regulation and function of PKB/AKT—A major therapeutic target. Biochim. Biophys. Acta (BBA)—Proteins Proteom..

[42] Burgering B.M., Coffer P.J. (1995). Protein kinase B (c-Akt) in phosphatidylinositol-3-OH kinase signal transduction [see comments]. Nature.

[43] Okano J., Gaslightwala I., Birnbaum M.J., Rustgi A.K., Nakagawa H. (2000). Akt/protein kinase B isoforms are differentially regulated by epidermal growth factor stimulation. J. Biol. Chem..

[44] Wahl M., Carpenter G. (1991). Selective phospholipase C activation. Bioessays.

[45] Kamat A., Carpenter G. (1997). Phospholipase C-gamma1: Regulation of enzyme function and role in growth factor-dependent signal transduction. Cytokine Growth Factor Rev..

[46] Wells A., Gupta K., Chang P., Swindle S., Glading A., Shiraha H. (1998). Epidermal growth factor receptor-mediated motility in fibroblasts. Microsc. Res. Tech..

[47] Gual P., Giordano S., Williams T.A., Rocchi S., Van Obberghen E., Comoglio P.M. (2000). Sustained recruitment of phospholipase C-gamma to Gab1 is required for HGF-induced branching tubulogenesis. Oncogene.

[48] Meyer R.D., Latz C., Rahimi N. (2003). Recruitment and activation of phospholipase Cgamma1 by vascular endothelial growth factor receptor-2 are required for tubulogenesis and differentiation of endothelial cells. J. Biol. Chem..

[49] Bivona T.G., de Castro I.P., Ahearn I.M., Grana T.M., Chiu V.K., Lockyer P.J., Cullen P.J., Pellicer A., Cox A.D., Philips M.R. (2003). Phospholipase Cgamma activates Ras on the Golgi apparatus by means of RasGRP1. Nature.

[50] Chou J., Burke N.A., Iwabu A., Watkins S.C., Wells A. (2003). Directional motility induced by epidermal growth factor requires Cdc42. Exp. Cell Res..

[51] Choi J.H., Park J.B., Bae S.S., Yun S., Kim H.S., Hong W.P., Kim I.S., Kim J.H., Han M.Y., Ryu S.H. (2004). Phospholipase C-gamma1 is a guanine nucleotide exchange factor for dynamin-1 and enhances dynamin-1-dependent epidermal growth factor receptor endocytosis. J. Cell Sci..

[52] Thomas S.M., Coppelli F.M., Wells A., Gooding W.E., Song J., Kassis J., Drenning S.D., Grandis J.R. (2003). Epidermal growth factor receptor-stimulated activation of phospholipase Cgamma-1 promotes invasion of head and neck squamous cell carcinoma. Cancer Res..

[53] Wells A., Grandis J.R. (2003). Phospholipase C-gamma1 in tumor progression. Clin. Exp. Metastasis.

[54] Wahl M.I., Daniel T.O., Carpenter G. (1988). Antiphosphotyrosine recovery of phospholipase C activity after EGF treatment of A-431 cells. Science.

[55] Margolis B., Rhee S.G., Felder S., Mervic M., Lyall R., Levitzki A., Ullrich A., Zilberstein A., Schlessinger J. (1989). EGF induces tyrosine phosphorylation of phospholipase C-II: A potential mechanism for EGF receptor signaling. Cell.

[56] Meisenhelder J., Suh P.G., Rhee S.G., Hunter T. (1989). Phospholipase C-gamma is a substrate for the PDGF and EGF receptor protein-tyrosine kinases in vivo and in vitro. Cell.

[57] Anderson D., Koch C.A., Grey L., Ellis C., Moran M.F., Pawson T. (1990). Binding of SH2 domains of phospholipase C gamma 1, GAP, and Src to activated growth factor receptors. Science.

[58] Margolis B., Zilberstein A., Franks C., Felder S., Kremer S., Ullrich A., Rhee S.G., Skorecki K., Schlessinger J. (1990). Effect of phospholipase C-gamma overexpression on PDGF-induced second messengers and mitogenesis. Science.

[59] Kim H.K., Kim J.W., Zilberstein A., Margolis B., Kim J.G., Schlessinger J., Rhee S.G. (1991). PDGF stimulation of inositol phospholipid hydrolysis requires PLC-gamma 1 phosphorylation on tyrosine residues 783 and 1254. Cell.

[60] Rotin D., Honegger A.M., Margolis B.L., Ullrich A., Schlessinger J. (1992). Presence of SH2 domains of phospholipase C gamma 1 enhances substrate phosphorylation by increasing the affinity toward the epidermal growth factor receptor. J. Biol. Chem..

[61] Ronnstrand L., Mori S., Arridsson A.K., Eriksson A., Wernstedt C., Hellman U., Claesson-Welsh L., Heldin C.H. (1992). Identification of two C-terminal autophosphorylation sites in the PDGF beta-receptor: Involvement in the interaction with phospholipase C- gamma. EMBO J..

[62] Quesnelle K.M., Boehm A.L., Grandis J.R. (2007). STAT-mediated EGFR signaling in cancer. J. Cell Biochem..

[63] Wang Z., Ceresa B. (2012). Mutual Regulation of Receptor-Mediated Cell Signalling and Endocytosis: EGF Receptor System as an Example. Molecular Regulation of Endocytosis.

[64] Ghosh C., Xing Y., Sun Y., Faber A.C. (2023). Chapter 6—Control of EGFR signaling by endocytosis and endosomal trafficking. Overcoming Resistance to EGFR Inhibitors in EGFR-Mutant NSCLC.

[65] Renard H.-F., Boucrot E. (2021). Unconventional endocytic mechanisms. Curr. Opin. Cell Biol..

[66] von Zastrow M., Sorkin A. (2021). Mechanisms for Regulating and Organizing Receptor Signaling by Endocytosis. Annu. Rev. Biochem..

[67] Perez Verdaguer M., Zhang T., Paulo J.A., Gygi S., Watkins S.C., Sakurai H., Sorkin A. (2021). Mechanism of p38 MAPK-induced EGFR endocytosis and its crosstalk with ligand-induced pathways. J. Cell Biol..

[68] Levkowitz G., Waterman H., Zamir E., Kam Z., Oved S., Langdon W.Y., Beguinot L., Geiger B., Yarden Y. (1998). c-Cbl/Sli-1 regulates endocytic sorting and ubiquitination of the epidermal growth factor receptor. Genes Dev..

[69] Levkowitz G., Waterman H., Ettenberg S.A., Katz M., Tsygankov A.Y., Alroy I., Lavi S., Iwai K., Reiss Y., Ciechanover A. (1999). Ubiquitin ligase activity and tyrosine phosphorylation underlie suppression of growth factor signaling by c-Cbl/Sli-1. Mol. Cell.

[70] Fukazawa T., Miyake S., Band V., Band H. (1996). Tyrosine phosphorylation of Cbl upon epidermal growth factor (EGF) stimulation and its association with EGF receptor and downstream signaling proteins. J. Biol. Chem..

[71] Joazeiro C.A., Wing S.S., Huang H., Leverson J.D., Hunter T., Liu Y.C. (1999). The tyrosine kinase negative regulator c-Cbl as a RING-type, E2-dependent ubiquitin-protein ligase. Science.

[72] Zheng N., Wang P., Jeffrey P.D., Pavletich N.P. (2000). Structure of a c-Cbl-UbcH7 complex: RING domain function in ubiquitin-protein ligases. Cell.

[73] Ravid T., Heidinger J.M., Gee P., Khan E.M., Goldkorn T. (2004). c-Cbl-mediated ubiquitinylation is required for epidermal growth factor receptor exit from the early endosomes. J. Biol. Chem..

[74] Haglund K., Sigismund S., Polo S., Szymkiewicz I., Di Fiore P.P., Dikic I. (2003). Multiple monoubiquitination of RTKs is sufficient for their endocytosis and degradation. Nat. Cell Biol..

[75] Huang F., Sorkin A. (2005). Growth factor receptor binding protein 2-mediated recruitment of the RING domain of Cbl to the epidermal growth factor receptor is essential and sufficient to support receptor endocytosis. Mol. Biol. Cell.

[76] Pennock S., Wang Z. (2008). A tale of two Cbls: Interplay of c-Cbl and Cbl-b in epidermal growth factor receptor downregulation. Mol. Cell. Biol..

[77] Boretto M., Geurts M.H., Gandhi S., Ma Z., Staliarova N., Celotti M., Lim S., He G.W., Millen R., Driehuis E. (2024). Epidermal growth factor receptor (EGFR) is a target of the tumor-suppressor E3 ligase FBXW7. Proc. Natl. Acad. Sci. USA.

[78] Hirata Y., Orth D.N. (1979). Epidermal growth factor (urogastrone) in human tissues. J. Clin. Endocrinol. Metab..

[79] Kirkham M., Parton R.G. (2005). Clathrin-independent endocytosis: New insights into caveolae and non-caveolar lipid raft carriers. Biochim. Biophys. Acta (BBA)—Mol. Cell Res..

[80] Doherty G.J., McMahon H.T. (2009). Mechanisms of endocytosis. Annu. Rev. Biochem..

[81] Johannes L. (2017). Endocytosis: Remote Control from Deep Inside. Curr. Biol..

[82] Liu L., Shi H., Chen X., Wang Z. (2011). Regulation of EGF-stimulated EGF receptor endocytosis during M phase. Traffic.

[83] Connolly J.M., Rose D.P. (1988). Epidermal growth factor-like proteins in breast fluid and human milk. Life Sci..

[84] Dvorak B. (2010). Milk epidermal growth factor and gut protection. J. Pediatr..

[85] Murdoch-Kinch C.A., Russo N., Griffith S., Braun T., Eisbruch A., D’Silva N.J. (2011). Recovery of salivary epidermal growth factor in parotid saliva following parotid sparing radiation therapy: A proof-of-principle study. Oral Surg. Oral Med. Oral Pathol. Oral Radiol. Endodontology.

[86] Fisher D.A., Lakshmanan J. (1990). Metabolism and effects of epidermal growth factor and related growth factors in mammals. Endocr. Rev..

[87] Schultz G., Rotatori D.S., Clark W. (1991). EGF and TGF-alpha in wound healing and repair. J. Cell. Biochem..

[88] Wee P., Wang Z. (2018). Regulation of EGFR Endocytosis by CBL During Mitosis. Cells.

[89] Wang Q., Zhu F., Wang Z. (2007). Identification of EGF Receptor C-terminal Sequences 1005–1017 and Di-leucine Motif ^1010^LL^1011^ as Essential in EGF Receptor Endocytosis. Exp.Cell Res..

[90] Wang Q., Chen X., Wang Z. (2015). Dimerization drives EGFR endocytosis through two sets of compatible endocytic codes. J. Cell Sci..

[91] Vergarajauregui S., San Miguel A., Puertollano R. (2006). Activation of p38 mitogen-activated protein kinase promotes epidermal growth factor receptor internalization. Traffic.

[92] Frey M.R., Dise R.S., Edelblum K.L., Polk D.B. (2006). p38 kinase regulates epidermal growth factor receptor downregulation and cellular migration. EMBO J..

[93] Zwang Y., Yarden Y. (2006). p38 MAP kinase mediates stress-induced internalization of EGFR: Implications for cancer chemotherapy. EMBO J..

[94] Zhou Y., Tanaka T., Sugiyama N., Yokoyama S., Kawasaki Y., Sakuma T., Ishihama Y., Saiki I., Sakurai H. (2014). p38-Mediated phosphorylation of Eps15 endocytic adaptor protein. FEBS Lett..

[95] Metz C., Oyanadel C., Jung J., Retamal C., Cancino J., Barra J., Venegas J., Du G., Soza A., González A. (2021). Phosphatidic acid-PKA signaling regulates p38 and ERK1/2 functions in ligand-independent EGFR endocytosis. Traffic.

[96] Kim E.H., Kim M.-S., Takahashi A., Suzuki M., Vares G., Uzawa A., Fujimori A., Ohno T., Sai S. (2020). Carbon-Ion Beam Irradiation Alone or in Combination with Zoledronic acid Effectively Kills Osteosarcoma Cells. Cancers.

[97] Tomas A., Futter C.E., Eden E.R. (2014). EGF receptor trafficking: Consequences for signaling and cancer. Trends Cell Biol..

[98] Zhou Y., Sakurai H. (2022). New trend in ligand-induced EGFR trafficking: A dual-mode clathrin-mediated endocytosis model. J. Proteom..

[99] Santos E.D.S., Nogueira K.A.B., Fernandes L.C.C., Martins J.R.P., Reis A.V.F., Neto J.B.V., Júnior I., Pessoa C., Petrilli R., Eloy J.O. (2021). EGFR targeting for cancer therapy: Pharmacology and immunoconjugates with drugs and nanoparticles. Int. J. Pharm..

[100] Takahashi J.-I., Nakamura S., Onuma I., Zhou Y., Yokoyama S., Sakurai H. (2022). Synchronous intracellular delivery of EGFR-targeted antibody–drug conjugates by p38-mediated non-canonical endocytosis. Sci. Rep..

[101] Lin S.Y., Makino K., Xia W., Matin A., Wen Y., Kwong K.Y., Bourguignon L., Hung M.C. (2001). Nuclear localization of EGF receptor and its potential new role as a transcription factor. Nat. Cell Biol..

[102] Dittmann K., Mayer C., Fehrenbacher B., Schaller M., Raju U., Milas L., Chen D.J., Kehlbach R., Rodemann H.P. (2005). Radiation-induced epidermal growth factor receptor nuclear import is linked to activation of DNA-dependent protein kinase. J. Biol. Chem..

[103] Hsu S.C., Hung M.C. (2007). Characterization of a novel tripartite nuclear localization sequence in the EGFR family. J. Biol. Chem..

[104] Giri D.K., Ali-Seyed M., Li L.Y., Lee D.F., Ling P., Bartholomeusz G., Wang S.C., Hung M.C. (2005). Endosomal transport of ErbB-2: Mechanism for nuclear entry of the cell surface receptor. Mol. Cell. Biol..

[105] Wang Y.N., Yamaguchi H., Hsu J.M., Hung M.C. (2010). Nuclear trafficking of the epidermal growth factor receptor family membrane proteins. Oncogene.

[106] Wang T.-H., Wu C.-C., Huang K.-Y., Chuang W.-Y., Hsueh C., Li H.-J., Chen C.-Y. (2020). Profiling of subcellular EGFR interactome reveals hnRNP A3 modulates nuclear EGFR localization. Oncogenesis.

[107] Zhu J., Wu Z., Shan G., Huang Y., Liang J., Zhan C. (2024). Nuclear epidermal growth factor receptor (nEGFR) in clinical treatment. Heliyon.

[108] De Angelis Campos A.C., Rodrigues M.A., de Andrade C., de Goes A.M., Nathanson M.H., Gomes D.A. (2011). Epidermal growth factor receptors destined for the nucleus are internalized via a clathrin-dependent pathway. Biochem. Biophys. Res. Commun..

[109] Wang Y.N., Wang H., Yamaguchi H., Lee H.J., Lee H.H., Hung M.C. (2010). COPI-mediated retrograde trafficking from the Golgi to the ER regulates EGFR nuclear transport. Biochem. Biophys. Res. Commun..

[110] Wang Y.-N., Yamaguchi H., Huo L., Du Y., Lee H.-J., Lee H.-H., Wang H., Hsu J.-M., Hung M.-C. (2010). The Translocon Sec61β Localized in the Inner Nuclear Membrane Transports Membrane-embedded EGF Receptor to the Nucleus. J. Biol. Chem..

[111] Brand T.M., Iida M., Li C., Wheeler D.L. (2011). The nuclear epidermal growth factor receptor signaling network and its role in cancer. Discov. Med..

[112] Demory M.L., Boerner J.L., Davidson R., Faust W., Miyake T., Lee I., Huttemann M., Douglas R., Haddad G., Parsons S.J. (2009). Epidermal growth factor receptor translocation to the mitochondria: Regulation and effect. J. Biol. Chem..

[113] Cao X., Zhu H., Ali-Osman F., Lo H.W. (2011). EGFR and EGFRvIII undergo stress- and EGFR kinase inhibitor-induced mitochondrial translocalization: A potential mechanism of EGFR-driven antagonism of apoptosis. Mol. Cancer.

[114] Wang T.H., Lin Y.H., Yang S.C., Chang P.C., Wang T.C., Chen C.Y. (2017). Tid1-S regulates the mitochondrial localization of EGFR in non-small cell lung carcinoma. Oncogenesis.

[115] Bollu L.R., Ren J., Blessing A.M., Katreddy R.R., Gao G., Xu L., Wang J., Su F., Weihua Z. (2014). Involvement of de novo synthesized palmitate and mitochondrial EGFR in EGF induced mitochondrial fusion of cancer cells. Cell Cycle.

[116] Che T.F., Lin C.W., Wu Y.Y., Chen Y.J., Han C.L., Chang Y.L., Wu C.T., Hsiao T.H., Hong T.M., Yang P.C. (2015). Mitochondrial translocation of EGFR regulates mitochondria dynamics and promotes metastasis in NSCLC. Oncotarget.

[117] Aghazadeh Y., Papadopoulos V. (2016). The role of the 14-3-3 protein family in health, disease, and drug development. Drug Discov. Today.

[118] Di Fiore P.P., Gill G.N. (1999). Endocytosis and mitogenic signaling. Curr. Opin. Cell Biol..

[119] Clague M.J., Urbe S. (2001). The interface of receptor trafficking and signalling. J. Cell Sci..

[120] Wang Y., Pennock S., Chen X., Wang Z. (2002). Endosomal signaling of epidermal growth factor receptor stimulates signal transduction pathways leading to cell survival. Mol. Cell. Biol..

[121] Pennock S., Wang Z. (2003). Stimulation of cell proliferation by endosomal epidermal growth factor receptor as revealed through two distinct phases of signaling. Mol. Cell. Biol..

[122] Dobrowolski R., De Robertis E.M. (2012). Endocytic control of growth factor signalling: Multivesicular bodies as signalling organelles. Nat. Rev. Mol. Cell. Biol..

[123] Carpenter G., Cohen S. (1979). Epidermal growth factor. Annu. Rev. Biochem..

[124] McKanna J.A., Haigler H.T., Cohen S. (1979). Hormone receptor topology and dynamics: Morphological analysis using ferritin-labeled epidermal growth factor. Proc. Natl. Acad. Sci. USA.

[125] Roepstorff K., Grovdal L., Grandal M., Lerdrup M., van Deurs B. (2008). Endocytic downregulation of ErbB receptors: Mechanisms and relevance in cancer. Histochem. Cell Biol..

[126] Garcia de Palazzo I.E., Adams G.P., Sundareshan P., Wong A.J., Testa J.R., Bigner D.D., Weiner L.M. (1993). Expression of mutated epidermal growth factor receptor by non-small cell lung carcinomas. Cancer Res..

[127] Moscatello D.K., Holgado-Madruga M., Emlet D.R., Montgomery R.B., Wong A.J. (1998). Constitutive activation of phosphatidylinositol 3-kinase by a naturally occurring mutant epidermal growth factor receptor. J. Biol. Chem..

[128] Ge H., Gong X., Tang C.K. (2002). Evidence of high incidence of EGFRvIII expression and coexpression with EGFR in human invasive breast cancer by laser capture microdissection and immunohistochemical analysis. Int. J. Cancer.

[129] Nishikawa R., Ji X.D., Harmon R.C., Lazar C.S., Gill G.N., Cavenee W.K., Huang H.J. (1994). A mutant epidermal growth factor receptor common in human glioma confers enhanced tumorigenicity. Proc. Natl. Acad. Sci. USA.

[130] Nagane M., Coufal F., Lin H., Bogler O., Cavenee W.K., Huang H.J. (1996). A common mutant epidermal growth factor receptor confers enhanced tumorigenicity on human glioblastoma cells by increasing proliferation and reducing apoptosis. Cancer Res..

[131] Pedersen M.W., Pedersen N., Ottesen L.H., Poulsen H.S. (2005). Differential response to gefitinib of cells expressing normal EGFR and the mutant EGFRvIII. Br. J. Cancer.

[132] Han W., Zhang T., Yu H., Foulke J.G., Tang C.K. (2006). Hypophosphorylation of residue Y1045 leads to defective downregulation of EGFRvIII. Cancer Biol. Ther..

[133] Cohen S., Fava R.A. (1985). Internalization of functional epidermal growth factor: Receptor/kinase complexes in A-431 cells. J. Biol. Chem..

[134] Kay D.G., Lai W.H., Uchihashi M., Khan M.N., Posner B.I., Bergeron J.J. (1986). Epidermal growth factor receptor kinase translocation and activation in vivo. J. Biol. Chem..

[135] Lai W.H., Cameron P.H., Doherty J.J., Posner B.I., Bergeron J.J. (1989). Ligand-mediated autophosphorylation activity of the epidermal growth factor receptor during internalization. J. Cell Biol..

[136] Di Guglielmo G.M., Baass P.C., Ou W.J., Posner B.I., Bergeron J.J. (1994). Compartmentalization of SHC, GRB2 and mSOS, and hyperphosphorylation of Raf-1 by EGF but not insulin in liver parenchyma. EMBO J..

[137] Wang Z., Tung P.S., Moran M.F. (1996). Association of p120 ras GAP with endocytic components and colocalization with epidermal growth factor (EGF) receptor in response to EGF stimulation. Cell Growth Differ..

[138] Haugh J.M., Huang A.C., Wiley H.S., Wells A., Lauffenburger D.A. (1999). Internalized epidermal growth factor receptors participate in the activation of p21(ras) in fibroblasts. J. Biol. Chem..

[139] Kuruvilla R., Ye H., Ginty D.D. (2000). Spatially and functionally distinct roles of the PI3-K effector pathway during NGF signaling in sympathetic neurons. Neuron.

[140] Vieira A.V., Lamaze C., Schmid S.L. (1996). Control of EGF receptor signaling by clathrin-mediated endocytosis. Science.

[141] Rea K., Sensi M., Anichini A., Canevari S., Tomassetti A. (2013). EGFR/MEK/ERK/CDK5-dependent integrin-independent FAK phosphorylated on serine 732 contributes to microtubule depolymerization and mitosis in tumor cells. Cell Death Dis..

[142] Uribe M.L., Marrocco I., Yarden Y. (2021). EGFR in Cancer: Signaling Mechanisms, Drugs, and Acquired Resistance. Cancers.

[143] Du Y., Karatekin F., Wang W.K., Hong W., Boopathy G.T.K. (2025). Cracking the EGFR code: Cancer biology, resistance mechanisms, and future therapeutic frontiers. Pharmacol. Rev..

[144] Franovic A., Gunaratnam L., Smith K., Robert I., Patten D., Lee S. (2007). Translational up-regulation of the EGFR by tumor hypoxia provides a nonmutational explanation for its overexpression in human cancer. Proc. Natl. Acad. Sci. USA.

[145] Hwang W., Ahn J.S., Jung H.A., Wirth L.J., Park J.C. (2025). Emerging EGFR-Targeted Therapy in Head and Neck Cancer: A Review. JAMA Oncol..

[146] Lassman A.B., Aldape K.D., Ansell P.J., Bain E., Curran W.J., Eoli M., French P.J., Kinoshita M., Looman J., Mehta M. (2019). Epidermal growth factor receptor (EGFR) amplification rates observed in screening patients for randomized trials in glioblastoma. J. Neurooncol..

[147] Jorissen R.N., Walker F., Pouliot N., Garrett T.P.J., Ward C.W., Burgess A.W. (2003). Epidermal growth factor receptor: Mechanisms of activation and signalling. Exp. Cell Res..

[148] Guo G., Gong K., Wohlfeld B., Hatanpaa K.J., Zhao D., Habib A.A. (2015). Ligand-Independent EGFR Signaling. Cancer Res..

[149] Randon G., Yaeger R., Hechtman J.F., Manca P., Fucà G., Walch H., Lee J., Élez E., Seligmann J., Mussolin B. (2021). EGFR Amplification in Metastatic Colorectal Cancer. J. Natl. Cancer Inst..

[150] Tavernari D., Borgeaud M., Liu X., Parikh K., Le X., Ciriello G., Addeo A. (2025). Decoding the Clinical and Molecular Signatures of EGFR Common, Compound, and Uncommon Mutations in NSCLC: A Brief Report. J. Thorac. Oncol..

[151] Greenall S.A., Johns T.G. (2016). EGFRvIII: The promiscuous mutation. Cell Death Discov..

[152] Su J., Zhong W., Zhang X., Huang Y., Yan H., Yang J., Dong Z., Xie Z., Zhou Q., Huang X. (2017). Molecular characteristics and clinical outcomes of EGFR exon 19 indel subtypes to EGFR TKIs in NSCLC patients. Oncotarget.

[153] Shigematsu H., Lin L., Takahashi T., Nomura M., Suzuki M., Wistuba I.I., Fong K.M., Lee H., Toyooka S., Shimizu N. (2005). Clinical and biological features associated with epidermal growth factor receptor gene mutations in lung cancers. J. Natl. Cancer Inst..

[154] Hsu W.H., Yang J.C.H., Mok T.S., Loong H.H. (2018). Overview of current systemic management of EGFR-mutant NSCLC. Ann. Oncol..

[155] Fabrizio F.P., Attili I., de Marinis F. (2024). Uncommon and Rare EGFR Mutations in Non-Small Cell Lung Cancer Patients with a Focus on Exon 20 Insertions and the Phase 3 PAPILLON Trial: The State of the Art. Cancers.

[156] Trinh J.Q., Abughanimeh O. (2024). Current management of uncommon EGFR mutations in non-small cell lung cancer. Curr. Probl. Cancer.

[157] Sehgal K., Rangachari D., VanderLaan P.A., Kobayashi S.S., Costa D.B. (2021). Clinical Benefit of Tyrosine Kinase Inhibitors in Advanced Lung Cancer with EGFR-G719A and Other Uncommon EGFR Mutations. Oncologist.

[158] Kobayashi Y., Togashi Y., Yatabe Y., Mizuuchi H., Jangchul P., Kondo C., Shimoji M., Sato K., Suda K., Tomizawa K. (2015). EGFR Exon 18 Mutations in Lung Cancer: Molecular Predictors of Augmented Sensitivity to Afatinib or Neratinib as Compared with First- or Third-Generation TKIs. Clin. Cancer Res..

[159] Park S., Lee S.Y., Kim D., Sim Y.S., Ryu J.S., Choi J., Lee S.H., Ryu Y.J., Lee J.H., Chang J.H. (2021). Comparison of epidermal growth factor receptor tyrosine kinase inhibitors for patients with lung adenocarcinoma harboring different epidermal growth factor receptor mutation types. BMC Cancer.

[160] Kitadai R., Okuma Y. (2022). Treatment Strategies for Non-Small Cell Lung Cancer Harboring Common and Uncommon EGFR Mutations: Drug Sensitivity Based on Exon Classification, and Structure-Function Analysis. Cancers.

[161] Vyse S., Huang P.H. (2019). Targeting EGFR exon 20 insertion mutations in non-small cell lung cancer. Signal Transduct. Target. Ther..

[162] Yu L., Wang R., Zhao Y., Wu Y., Wang L., Chen H., He Z., Wang Q., Wu Y. (2023). Advances in the Treatment of Rare Epidermal Growth Factor Receptor Mutations in Advanced Nonsmall-Cell Lung Cancer. Technol. Cancer Res. Treat..

[163] Meador C.B., Sequist L.V., Piotrowska Z. (2021). Targeting EGFR Exon 20 Insertions in Non-Small Cell Lung Cancer: Recent Advances and Clinical Updates. Cancer Discov..

[164] Jia Y., Yun C.H., Park E., Ercan D., Manuia M., Juarez J., Xu C., Rhee K., Chen T., Zhang H. (2016). Overcoming EGFR(T790M) and EGFR(C797S) resistance with mutant-selective allosteric inhibitors. Nature.

[165] Zhao B., Xiao Z., Qi J., Luo R., Lan Z., Zhang Y., Hu X., Tang Q., Zheng P., Xu S. (2019). Design, synthesis and biological evaluation of AZD9291 derivatives as selective and potent EGFRL858R/T790M inhibitors. Eur. J. Med. Chem..

[166] Gan H.K., Cvrljevic A.N., Johns T.G. (2013). The epidermal growth factor receptor variant III (EGFRvIII): Where wild things are altered. FEBS J..

[167] Dean L., Kane M., Pratt V.M., Scott S.A., Pirmohamed M., Esquivel B., Kattman B.L., Malheiro A.J. (2012). Cetuximab Therapy and RAS and BRAF Genotype. MINI Medical Genetics Summaries.

[168] Nickerson N.K., Mill C.P., Wu H.J., Riese D.J., Foley J. (2013). Autocrine-derived epidermal growth factor receptor ligands contribute to recruitment of tumor-associated macrophage and growth of basal breast cancer cells in vivo. Oncol. Res..

[169] Schrevel M., Osse E.M., Prins F.A., Trimbos J., Fleuren G.J., Gorter A., Jordanova E.S. (2017). Autocrine expression of the epidermal growth factor receptor ligand heparin-binding EGF-like growth factor in cervical cancer. Int. J. Oncol..

[170] Chakraborty S., Li L., Puliyappadamba V.T., Guo G., Hatanpaa K.J., Mickey B., Souza R.F., Vo P., Herz J., Chen M.R. (2014). Constitutive and ligand-induced EGFR signalling triggers distinct and mutually exclusive downstream signalling networks. Nat. Commun..

[171] Carpenter B.L., Chen M., Knifley T., Davis K.A., Harrison S.M.W., Stewart R.L., O’Connor K.L. (2015). Integrin α6β4 Promotes Autocrine Epidermal Growth Factor Receptor (EGFR) Signaling to Stimulate Migration and Invasion toward Hepatocyte Growth Factor (HGF). J. Biol. Chem..

[172] Yonesaka K., Zejnullahu K., Okamoto I., Satoh T., Cappuzzo F., Souglakos J., Ercan D., Rogers A., Roncalli M., Takeda M. (2011). Activation of ERBB2 signaling causes resistance to the EGFR-directed therapeutic antibody cetuximab. Sci. Transl. Med..

[173] Ungefroren H. (2021). Autocrine TGF-β in Cancer: Review of the Literature and Caveats in Experimental Analysis. Int. J. Mol. Sci..

[174] Austin C.D., De Maziere A.M., Pisacane P.I., van Dijk S.M., Eigenbrot C., Sliwkowski M.X., Klumperman J., Scheller R.H. (2004). Endocytosis and sorting of ErbB2 and the site of action of cancer therapeutics trastuzumab and geldanamycin. Mol. Biol. Cell.

[175] Wang Z., Zhang L., Yeung T.K., Chen X. (1999). Endocytosis deficiency of epidermal growth factor (EGF) receptor-ErbB2 heterodimers in response to EGF stimulation. Mol. Biol. Cell.

[176] Citri A., Yarden Y. (2006). EGF-ERBB signalling: Towards the systems level. Nat. Rev. Mol. Cell. Biol..

[177] Gong C., Zhang J., Zhang L., Wang Y., Ma H., Wu W., Cui J., Wang Y., Ren Z. (2015). Dynamin2 downregulation delays EGFR endocytic trafficking and promotes EGFR signaling and invasion in hepatocellular carcinoma. Am. J. Cancer Res..

[178] Al-Akhrass H., Naves T., Vincent F., Magnaudeix A., Durand K., Bertin F., Melloni B., Jauberteau M.O., Lalloué F. (2017). Sortilin limits EGFR signaling by promoting its internalization in lung cancer. Nat. Commun..

[179] Lapeyronnie E., Granet C., Tricard J., Gallet F., Yassine M., Daverat H., Rovini A., Chermat A., Jauberteau M.O., Bertin F. (2026). Sortilin exhibits tumor suppressor-like activity by limiting EGFR transduction function. Oncogene.

[180] Dang C.V. (2012). MYC on the path to cancer. Cell.

[181] Acunzo M., Romano G., Palmieri D., Laganá A., Garofalo M., Balatti V., Drusco A., Chiariello M., Nana-Sinkam P., Croce C.M. (2013). Cross-talk between MET and EGFR in non-small cell lung cancer involves miR-27a and Sprouty2. Proc. Natl. Acad. Sci. USA.

[182] Baker A.T., Zlobin A., Osipo C. (2014). Notch-EGFR/HER2 Bidirectional Crosstalk in Breast Cancer. Front. Oncol..

[183] Linklater E.S., Tovar E.A., Essenburg C.J., Turner L., Madaj Z., Winn M.E., Melnik M.K., Korkaya H., Maroun C.R., Christensen J.G. (2016). Targeting MET and EGFR crosstalk signaling in triple-negative breast cancers. Oncotarget.

[184] Hsu J.L., Hung M.C. (2016). The role of HER2, EGFR, and other receptor tyrosine kinases in breast cancer. Cancer Metastasis Rev..

[185] Cheng X. (2024). A Comprehensive Review of HER2 in Cancer Biology and Therapeutics. Genes.

[186] Lehmann B.D., Bauer J.A., Chen X., Sanders M.E., Chakravarthy A.B., Shyr Y., Pietenpol J.A. (2011). Identification of human triple-negative breast cancer subtypes and preclinical models for selection of targeted therapies. J. Clin. Investig..

[187] Hu T., Li C. (2010). Convergence between Wnt-beta-catenin and EGFR signaling in cancer. Mol. Cancer.

[188] Faivre E.J., Lange C.A. (2007). Progesterone receptors upregulate Wnt-1 to induce epidermal growth factor receptor transactivation and c-Src-dependent sustained activation of Erk1/2 mitogen-activated protein kinase in breast cancer cells. Mol. Cell. Biol..

[189] Tan X., Apte U., Micsenyi A., Kotsagrelos E., Luo J.H., Ranganathan S., Monga D.K., Bell A., Michalopoulos G.K., Monga S.P. (2005). Epidermal growth factor receptor: A novel target of the Wnt/beta-catenin pathway in liver. Gastroenterology.

[190] Suzuki M., Shigematsu H., Nakajima T., Kubo R., Motohashi S., Sekine Y., Shibuya K., Iizasa T., Hiroshima K., Nakatani Y. (2007). Synchronous alterations of Wnt and epidermal growth factor receptor signaling pathways through aberrant methylation and mutation in non small cell lung cancer. Clin. Cancer Res. Off. J. Am. Assoc. Cancer Res..

[191] Pancewicz-Wojtkiewicz J. (2016). Epidermal growth factor receptor and notch signaling in non-small-cell lung cancer. Cancer Med..

[192] Fu W., Li G., Lei C., Qian K., Zhang S., Zhao J., Hu S. (2023). Bispecific antibodies targeting EGFR/Notch enhance the response to talazoparib by decreasing tumour-initiating cell frequency. Theranostics.

[193] Liu F., Hon G.C., Villa G.R., Turner K.M., Ikegami S., Yang H., Ye Z., Li B., Kuan S., Lee A.Y. (2015). EGFR Mutation Promotes Glioblastoma through Epigenome and Transcription Factor Network Remodeling. Mol. Cell.

[194] Gondos A., Paz-Ares L.G., Saldana D., Thomas M., Mascaux C., Bubendorf L., Barlesi F. (2020). Genomic testing among patients (pts) with newly diagnosed advanced non-small cell lung cancer (aNSCLC) in the United States: A contemporary clinical practice patterns study. J. Clin. Oncol..

[195] Yao X., Gao C., Sun C., Chen Z.-S., Zhuang J. (2025). Epigenetic code underlying EGFR-TKI resistance in non-small cell lung cancer: Elucidation of mechanisms and perspectives on therapeutic strategies. Drug Discov. Today.

[196] Chou R.H., Wang Y.N., Hsieh Y.H., Li L.Y., Xia W., Chang W.C., Chang L.C., Cheng C.C., Lai C.C., Hsu J.L. (2014). EGFR modulates DNA synthesis and repair through Tyr phosphorylation of histone H4. Dev. Cell.

[197] Fabrizio F.P., Sparaneo A., Muscarella L.A. (2023). Monitoring EGFR-lung cancer evolution: A possible beginning of a “methylation era” in TKI resistance prediction. Front. Oncol..

[198] Cui F., Zhang X. (2021). Correlation between the overexpression of epidermal growth factor receptor and pathological features of gastric cancer: A meta-analysis. Transl. Cancer Res..

[199] Wang K.C., Chang H.Y. (2018). Epigenomics: Technologies and Applications. Circ. Res..

[200] Dickerson H., Diab A., Al Musaimi O. (2024). Epidermal Growth Factor Receptor Tyrosine Kinase Inhibitors in Cancer: Current Use and Future Prospects. Int. J. Mol. Sci..

[201] Wang Q., Zhu Y., Pei J. (2024). Targeting EGFR with molecular degraders as a promising strategy to overcome resistance to EGFR inhibitors. Future Med. Chem..

[202] Qiao D., Wang R.C., Wang Z. (2025). Precision Oncology: Current Landscape, Emerging Trends, Challenges, and Future Perspectives. Cells.

[203] Tan X., Thapa N., Sun Y., Anderson R.A. (2015). A kinase-independent role for EGF receptor in autophagy initiation. Cell.

[204] Eck M.J., Yun C.H. (2010). Structural and mechanistic underpinnings of the differential drug sensitivity of EGFR mutations in non-small cell lung cancer. Biochim. Biophys. Acta (BBA)—Proteins Proteom..

[205] Westover D., Zugazagoitia J., Cho B.C., Lovly C.M., Paz-Ares L. (2018). Mechanisms of acquired resistance to first- and second-generation EGFR tyrosine kinase inhibitors. Ann. Oncol. Off. J. Eur. Soc. Med. Oncol..

[206] Sos M.L., Rode H.B., Heynck S., Peifer M., Fischer F., Klüter S., Pawar V.G., Reuter C., Heuckmann J.M., Weiss J. (2010). Chemogenomic profiling provides insights into the limited activity of irreversible EGFR Inhibitors in tumor cells expressing the T790M EGFR resistance mutation. Cancer Res..

[207] Shirley M. (2018). Dacomitinib: First Global Approval. Drugs.

[208] Remon J., Steuer C.E., Ramalingam S.S., Felip E. (2018). Osimertinib and other third-generation EGFR TKI in EGFR-mutant NSCLC patients. Ann. Oncol. Off. J. Eur. Soc. Med. Oncol..

[209] Corvaja C., Passaro A., Attili I., Aliaga P.T., Spitaleri G., Signore E.D., de Marinis F. (2024). Advancements in fourth-generation EGFR TKIs in EGFR-mutant NSCLC: Bridging biological insights and therapeutic development. Cancer Treat. Rev..

[210] Su C., Sun S.Y. (2024). Fourth-generation epidermal growth factor receptor-tyrosine kinases inhibitors: Hope and challenges. Transl. Cancer Res..

[211] Cai W.Q., Zeng L.S., Wang L.F., Wang Y.Y., Cheng J.T., Zhang Y., Han Z.W., Zhou Y., Huang S.L., Wang X.W. (2020). The Latest Battles Between EGFR Monoclonal Antibodies and Resistant Tumor Cells. Front. Oncol..

[212] Battaglin F., Dadduzio V., Bergamo F., Manai C., Schirripa M., Lonardi S., Zagonel V., Loupakis F. (2017). Anti-EGFR monoclonal antibody panitumumab for the treatment of patients with metastatic colorectal cancer: An overview of current practice and future perspectives. Expert Opin. Biol. Ther..

[213] Sartore-Bianchi A., Pietrantonio F., Lonardi S., Mussolin B., Rua F., Crisafulli G., Bartolini A., Fenocchio E., Amatu A., Manca P. (2022). Circulating tumor DNA to guide rechallenge with panitumumab in metastatic colorectal cancer: The phase 2 CHRONOS trial. Nat. Med..

[214] Napolitano S., De Falco V., Martini G., Ciardiello D., Martinelli E., Della Corte C.M., Esposito L., Famiglietti V., Di Liello A., Avallone A. (2023). Panitumumab Plus Trifluridine-Tipiracil as Anti-Epidermal Growth Factor Receptor Rechallenge Therapy for Refractory RAS Wild-Type Metastatic Colorectal Cancer: A Phase 2 Randomized Clinical Trial. JAMA Oncol..

[215] Díaz-Serrano A., Sánchez-Torre A., Paz-Ares L. (2019). Necitumumab for the treatment of advanced non-small-cell lung cancer. Future Oncol..

[216] Takeda M., Okamoto I., Nishimura Y., Nakagawa K. (2011). Nimotuzumab, a novel monoclonal antibody to the epidermal growth factor receptor, in the treatment of non-small cell lung cancer. Lung Cancer Targets Ther..

[217] Zhao S., Cheng Y., Wang Q., Li X., Liao J., Rodon J., Meng X., Luo Y., Chen Z., Wang W. (2025). Sacituzumab tirumotecan in advanced non-small-cell lung cancer with or without EGFR mutations: Phase 1/2 and phase 2 trials. Nat. Med..

[218] Shang X., Zhang Z., Wu T., Nan Y., Ju D. (2026). EGFR-targeted therapies based on antibody engineering and nanotechnology for colorectal cancer. Biomaterials.

[219] Jaramillo M.L., Leon Z., Grothe S., Paul-Roc B., Abulrob A., O’Connor McCourt M. (2006). Effect of the anti-receptor ligand-blocking 225 monoclonal antibody on EGF receptor endocytosis and sorting. Exp. Cell Res..

[220] Liao C., Sun Q., Liang B., Shen J., Shuai X. (2011). Targeting EGFR-overexpressing tumor cells using Cetuximab-immunomicelles loaded with doxorubicin and superparamagnetic iron oxide. Eur. J. Radiol..

[221] Chen Y., Liu G., Guo L., Wang H., Fu Y., Luo Y. (2015). Enhancement of tumor uptake and therapeutic efficacy of EGFR-targeted antibody cetuximab and antibody-drug conjugates by cholesterol sequestration. Int. J. Cancer.

[222] Fukuya A., Sato Y., Okada Y., Sagawa T., Kawano Y., Bando M., Okamoto K., Miyamoto H., Sogabe M., Takayama T. (2026). UBR4 enhances efficacy of anti-EGFR antibody by facilitating clathrin-dependent EGFR endocytosis in colorectal cancer. J. Gastroenterol..

[223] Kiss A.L., Botos E. (2009). Endocytosis via caveolae: Alternative pathway with distinct cellular compartments to avoid lysosomal degradation?. J. Cell. Mol. Med..

[224] Hammood M., Craig A.W., Leyton J.V. (2021). Impact of Endocytosis Mechanisms for the Receptors Targeted by the Currently Approved Antibody-Drug Conjugates (ADCs)—A Necessity for Future ADC Research and Development. Pharmaceuticals.

[225] Zhuang X., Lin L., Li J., Yu G., Chen B., Xu Y., Chen S., Yi W., Pan L. (2026). Receptor-ubiquitination-targeting antibody-drug conjugates for enhanced endocytosis and lysosomal delivery. Cell Chem. Biol..

[226] Liu Y., Liu Y., Wu T., Bai X., Wang S., Zhang L., Fu Z., Shi C. (2026). Drug resistance to antibody-drug conjugates: Mechanisms, challenges, and perspectives. Cancer Biol. Med..

[227] He K., Xu J., Liang J., Jiang J., Tang M., Ye X., Zhang Z., Zhang L., Fu B., Li Y. (2019). Discovery of A Novel EGFR-Targeting Antibody-Drug Conjugate, SHR-A1307, for the Treatment of Solid Tumors Resistant or Refractory to Anti-EGFR Therapies. Mol. Cancer Ther..

[228] Li J.-H., Liu L., Zhao X.-H. (2024). Precision targeting in oncology: The future of conjugated drugs. Biomed. Pharmacother..

[229] Gomes-da-Silva L.C., Kepp O., Kroemer G. (2020). Regulatory approval of photoimmunotherapy: Photodynamic therapy that induces immunogenic cell death. Oncoimmunology.

[230] Deng S., Sun C., Liu D., Zhang Y., Zhang J., Li X., Jia X., Kai G. (2026). Antibody-drug conjugates: A new twist to overcome EGFR-TKIs resistance in non-small cell lung cancer. Pharmacol. Res..

[231] Gan H.K., Reardon D.A., Lassman A.B., Merrell R., van den Bent M., Butowski N., Lwin Z., Wheeler H., Fichtel L., Scott A.M. (2018). Safety, pharmacokinetics, and antitumor response of depatuxizumab mafodotin as monotherapy or in combination with temozolomide in patients with glioblastoma. Neuro-Oncology.

[232] Lassman A.B., Pugh S.L., Wang T.J.C., Aldape K., Gan H.K., Preusser M., Vogelbaum M.A., Sulman E.P., Won M., Zhang P. (2023). Depatuxizumab mafodotin in EGFR-amplified newly diagnosed glioblastoma: A phase III randomized clinical trial. Neuro-Oncology.

[233] Rotow J.K., Waqar S.N., Papadopoulos K.P., Hung J.Y., Spira A.I., Gan H., Yoshida T., Kuo C.H.S., Doger de Spéville B., Felip E. (2023). 1318MO First-in-human study of ABBV-637, an EGFR-targeting BCL-XL–inhibiting antibody-drug conjugate combined with osimertinib (OSI) in relapsed/refractory, EGFR-mutated non-small cell lung cancer (NSCLC). Ann. Oncol..

[234] Thwaites M.J., Figueredo R., Tremblay G., Koropatnick J., Goldmacher V., O’Connor-McCourt M. (2019). Abstract 218: AVID100 is an anti-EGFR ADC that promotes DM1-meditated cytotoxicity on cancer cells but not on normal cells. Cancer Res..

[235] Qiu M.Z., Zhang Y., Guo Y., Guo W., Nian W., Liao W., Xu Z., Zhang W., Zhao H.Y., Wei X. (2022). Evaluation of Safety of Treatment With Anti-Epidermal Growth Factor Receptor Antibody Drug Conjugate MRG003 in Patients With Advanced Solid Tumors: A Phase 1 Nonrandomized Clinical Trial. JAMA Oncol..

[236] Haikala H.M., Lopez T., Köhler J., Eser P.O., Xu M., Zeng Q., Teceno T.J., Ngo K., Zhao Y., Ivanova E.V. (2022). EGFR Inhibition Enhances the Cellular Uptake and Antitumor-Activity of the HER3 Antibody-Drug Conjugate HER3-DXd. Cancer Res..

[237] Kato Y., Kato Y., Minegishi Y., Suzuki T., Nakamichi S., Matsumoto M., Miyanaga A., Noro R., Kubota K., Terasaki Y. (2021). Efficacy with Trastuzumab Deruxtecan for Non-Small-Cell Lung Cancer Harboring HER2 Exon 20 Insertion Mutation in a Patient with a Poor Performance Status: A Case Report. OncoTargets Ther..

[238] Yu H.A., Baik C., Kim D.W., Johnson M.L., Hayashi H., Nishio M., Yang J.C., Su W.C., Gold K.A., Koczywas M. (2024). Translational insights and overall survival in the U31402-A-U102 study of patritumab deruxtecan (HER3-DXd) in EGFR-mutated NSCLC. Ann. Oncol. Off. J. Eur. Soc. Med. Oncol..

[239] Scott S., Levy B. (2024). New ADCs bring new questions in EGFR NSCLC and beyond. Ann. Oncol..

[240] Mamot C., Wicki A., Hasler-Strub U., Riniker S., Li Q., Holer L., Bärtschi D., Zaman K., von Moos R., Dedes K.J. (2023). A multicenter phase II trial of anti-EGFR-immunoliposomes loaded with doxorubicin in patients with advanced triple negative breast cancer. Sci. Rep..

[241] Crintea A., Constantin A.M., Motofelea A.C., Crivii C.B., Velescu M.A., Coșeriu R.L., Ilyés T., Crăciun A.M., Silaghi C.N. (2023). Targeted EGFR Nanotherapy in Non-Small Cell Lung Cancer. J. Funct. Biomater..

[242] Brown M.K., Cai Z., Ganga-Sah Y., Alshafai L., Borlase S., Radchenko V., Gao A., Rutka J.T., Reilly R.M. (2026). Treatment of Human Glioblastoma Multiforme in NRG Mice by Convection-Enhanced Delivery of EGFR-Targeted or Nontargeted Auger Electron-Emitting (197g)Hg-Labeled Gold Nanoparticles. J. Nucl. Med. Off. Publ. Soc. Nucl. Med..

[243] Kang S.J., Jeong H.Y., Kim M.W., Jeong I.H., Choi M.J., You Y.M., Im C.S., Song I.H., Lee T.S., Park Y.S. (2018). Anti-EGFR lipid micellar nanoparticles co-encapsulating quantum dots and paclitaxel for tumor-targeted theranosis. Nanoscale.

[244] Lee D.H., Choi K., Lee J.W., Kim M.W., Park Y.S., Choi M.J. (2026). Anti-EGFR Aptamer-Conjugated Erythrocyte Membrane-Derived Nanoparticles for Targeted Doxorubicin Delivery. Mol. Pharm..

[245] Moreira T.D.S., Silva A.D.O., Vasconcelos B.R.F., Santos E.D.S., de Sousa A.C.C., de Freitas J.V.B., de Oliveira Y.S., Vidal L.M.T., Ribeiro F.O.S., de Araújo A.R. (2023). DOPE/CHEMS-Based EGFR-Targeted Immunoliposomes for Docetaxel Delivery: Formulation Development, Physicochemical Characterization and Biological Evaluation on Prostate Cancer Cells. Pharmaceutics.

[246] Aggarwal S., Yadav S., Gupta S. (2011). EGFR targeted PLGA nanoparticles using gemcitabine for treatment of pancreatic cancer. J. Biomed. Nanotechnol..

[247] Deepagan V.G., Sarmento B., Menon D., Nascimento A., Jayasree A., Sreeranganathan M., Koyakutty M., Nair S.V., Rangasamy J. (2012). In vitro targeted imaging and delivery of camptothecin using cetuximab-conjugated multifunctional PLGA-ZnS nanoparticles. Nanomedicine.

[248] Jin H., Pi J., Zhao Y., Jiang J., Li T., Zeng X., Yang P., Evans C.E., Cai J. (2017). EGFR-targeting PLGA-PEG nanoparticles as a curcumin delivery system for breast cancer therapy. Nanoscale.

[249] Venugopal V., Krishnan S., Palanimuthu V.R., Sankarankutty S., Kalaimani J.K., Karupiah S., Kit N.S., Hock T.T. (2018). Anti-EGFR anchored paclitaxel loaded PLGA nanoparticles for the treatment of triple negative breast cancer. In-vitro and in-vivo anticancer activities. PLoS ONE.

[250] Bhattacharya S. (2021). Anti-EGFR-mAb and 5-Fluorouracil Conjugated Polymeric Nanoparticles for Colorectal Cancer. Recent Pat. Anti-Cancer Drug Discov..

[251] Salehnia Z., Shahbazi-Gahrouei D., Akbarzadeh A., Baradaran B., Farajnia S., Naghibi M. (2019). Synthesis and characterisation of iron oxide nanoparticles conjugated with epidermal growth factor receptor (EGFR) monoclonal antibody as MRI contrast agent for cancer detection. IET Nanobiotechnol..

[252] Gao J., Xia Y., Chen H., Yu Y., Song J., Li W., Qian W., Wang H., Dai J., Guo Y. (2014). Polymer-lipid hybrid nanoparticles conjugated with anti-EGF receptor antibody for targeted drug delivery to hepatocellular carcinoma. Nanomedicine.

[253] Fang F., Zhang X., Tang J., Wang Y., Xu J., Sun Y. (2023). EGFR-targeted hybrid lipid nanoparticles for chemo-photothermal therapy against colorectal cancer cells. Chem. Phys. Lipids.

[254] Haeri A., Zalba S., Ten Hagen T.L., Dadashzadeh S., Koning G.A. (2016). EGFR targeted thermosensitive liposomes: A novel multifunctional platform for simultaneous tumor targeted and stimulus responsive drug delivery. Colloids Surf. B Biointerfaces.

[255] Dorjsuren B., Chaurasiya B., Ye Z., Liu Y., Li W., Wang C., Shi D., Evans C.E., Webster T.J., Shen Y. (2020). Cetuximab-Coated Thermo-Sensitive Liposomes Loaded with Magnetic Nanoparticles and Doxorubicin for Targeted EGFR-Expressing Breast Cancer Combined Therapy. Int. J. Nanomed..

[256] Qian Y., Qiu M., Wu Q., Tian Y., Zhang Y., Gu N., Li S., Xu L., Yin R. (2014). Enhanced cytotoxic activity of cetuximab in EGFR-positive lung cancer by conjugating with gold nanoparticles. Sci. Rep..

[257] Morita M., Kang M.S., Choi J.H., Kim H., Nah S., Kwon S.-H., Lee R., Park Y.I. (2025). EGFR-targeted micelles-in-lipopolymersome nanocarriers for overcoming drug resistance in triple-negative breast cancer. J. Mater. Chem. B.

[258] Kumari L., Ehsan I., Mondal A., Al Hoque A., Mukherjee B., Choudhury P., Sengupta A., Sen R., Ghosh P. (2023). Cetuximab-conjugated PLGA nanoparticles as a prospective targeting therapeutics for non-small cell lung cancer. J. Drug Target..

[259] Otsuyama K., Morita S., Sato M., Zhou Y., Yokoyama S., Azuma K., Kato Y., Hayashi R., Sakurai H. (2026). Enhanced Efficacy of EGFR-Targeted ADCs via p38-Mediated Noncanonical Endocytosis in EGFR-Mutant Lung Cancer Cells. Biol. Pharm. Bull..

[260] Seo S.Y., Joo S.H., Lee S.O., Yoon G., Cho S.S., Choi Y.H., Park J.W., Shim J.H. (2024). Activation of p38 and JNK by ROS Contributes to Deoxybouvardin-Mediated Intrinsic Apoptosis in Oxaliplatin-Sensitive and -Resistant Colorectal Cancer Cells. Antioxidants.

[261] Haryuni R.D., Tanaka T., Takahashi J.I., Onuma I., Zhou Y., Yokoyama S., Sakurai H. (2021). Temozolomide Induces Endocytosis of EGFRvIII via p38-Mediated Non-canonical Phosphorylation in Glioblastoma Cells. Biol. Pharm. Bull..

[262] Cruz Da Silva E., Choulier L., Thevenard-Devy J., Schneider C., Carl P., Rondé P., Dedieu S., Lehmann M. (2021). Role of Endocytosis Proteins in Gefitinib-Mediated EGFR Internalisation in Glioma Cells. Cells.

[263] D’Ancona C., Cruz Da Silva E., Denechere L., Justiniano H., Calco V., Richert L., Vauchelles R., Sensoy D., Dussoulliez C., Seguin C. (2026). Exploring gefitinib to enhance endocytosis of antibodies and nucleic acid aptamers targeting EGFR in glioblastoma. Nanoscale.

[264] Blandin A.F., Cruz Da Silva E., Mercier M.C., Glushonkov O., Didier P., Dedieu S., Schneider C., Devy J., Etienne-Selloum N., Dontenwill M. (2021). Gefitinib induces EGFR and α5β1 integrin co-endocytosis in glioblastoma cells. Cell. Mol. Life Sci..

[265] Nishimura Y., Itoh K. (2019). Involvement of SNX1 in regulating EGFR endocytosis in a gefitinib-resistant NSCLC cell lines. Cancer Drug Resist..

[266] Kim B., Park Y.S., Sung J.S., Lee J.W., Lee S.B., Kim Y.H. (2021). Clathrin-mediated EGFR endocytosis as a potential therapeutic strategy for overcoming primary resistance of EGFR TKI in wild-type EGFR non-small cell lung cancer. Cancer Med..

[267] Acharya S., Dilnawaz F., Sahoo S.K. (2009). Targeted epidermal growth factor receptor nanoparticle bioconjugates for breast cancer therapy. Biomaterials.

[268] Ren K., Gong H., Ma Z., Tian L., Ye W., Lv X., Wu C. (2022). Structure and activity of an anti-epidermal growth factor receptor antibody without galactose-α-1,3-galactose residues. Drug Dev. Res..

[269] Pacholczak-Madej R., Meluch M., Püsküllüoğlu M. (2025). Sequencing of antibody drug conjugates in breast cancer: Evidence gap and future directions. Biochim. Biophys. Acta (BBA)—Rev. Cancer.

[270] Klein C., Brinkmann U., Reichert J.M., Kontermann R.E. (2024). The present and future of bispecific antibodies for cancer therapy. Nat. Rev. Drug Discov..

[271] Liu C., Liu D., Ji Y., Sun M., Gao S., Ma X., Zhong D., Zhu J., Cao Y., Qi C. (2025). A bispecific antibody–drug conjugate targeting EGFR and HER3 in metastatic esophageal squamous cell carcinoma: A phase 1b trial. Nat. Med..

[272] Maron S.B., Tsai C., Strickland M., Chou J.F., Roomi S.R.S., Vanderbilt C., Tang L.H., Srivastava A., Gu P., Joshi S.S. (2026). AMIGO: A phase 2 trial of amivantamab in *EGFR* or *MET*-amplified gastroesophageal adenocarcinoma (GEA). J. Clin. Oncol..

[273] Guo H., Wang Y., Diao M., Han R., He T., Wu Y., Wang Q., Cheng L., Zhao C., Li X. (2026). Bispecific T-cell Engager (CD3 × EGFR)-Based Triplet Therapy Unlocks CD40/CD40L Crosstalk to Revert Immunosuppression in Third-Generation EGFR-Tyrosine Kinase Inhibitor–Refractory NSCLC. J. Thorac. Oncol..

[274] Yu S., Zheng Y., Jia J., Kong X., Wang J., Luo Y., Ye H., Ma X., Yang Q., Zhao R.Y. (2026). Abstract 2394: A novel 2 + 2 IgG-like bispecific antibody-drug conjugate targeting EBV GP220/350 and EGFR for selective treatment of nasopharyngeal carcinoma. Cancer Res..

[275] Kühl L., Löffler A.K., Seifert O., Michler D., Kuhlmann M., Russo G., Kuhn P., Nowack H., Frenzel A., Schirrmann T. (2026). Novel bispecific T-cell engagers overcoming acquired EGFR resistance. mAbs.

[276] Boustany L.M., LaPorte S.L., Wong L., White C., Vinod V., Shen J., Yu W., Koditek D., Winter M.B., Moore S.J. (2022). A Probody T Cell-Engaging Bispecific Antibody Targeting EGFR and CD3 Inhibits Colon Cancer Growth with Limited Toxicity. Cancer Res..

[277] Verhaar E.R., Woodham A.W., Ploegh H.L. (2021). Nanobodies in cancer. Semin. Immunol..

[278] Heitrich M., Fernandez M.M., Aguilar-Cortes D.C., Werbajh S., Canziani G., Zylberman V., Ripari L.B., Videla-Richardson G.A., Malchiodi E.L., Podhajcer O.L. (2025). Development of novel high-affinity nanobodies against EGFR for cancer therapy. bioRxiv.

[279] Chen B., Zheng X., Wu J., Chen G., Yu J., Xu Y., Wu W.K.K., Tse G.M.K., To K.F., Kang W. (2025). Antibody-drug conjugates in cancer therapy: Current landscape, challenges, and future directions. Mol. Cancer.

[280] Habibi S., Bahramian S., Zare Jalise S., Mehri S., Ababzadeh S., Kavianpour M. (2025). Novel strategies in breast cancer management: From treatment to long-term remission. Crit. Rev. Oncol./Hematol..

[281] Stumpo S., Carlini A., Mantuano F., Di Federico A., Melotti B., Sperandi F., Favorito V., De Giglio A. (2025). Efficacy and Safety of TROP-2-Targeting Antibody-Drug Conjugate Treatment in Previously Treated Patients with Advanced Non-Small Cell Lung Cancer: A Systematic Review and Pooled Analysis of Reconstructed Patient Data. Cancers.

[282] Parveen I., Aqil A., Sharma S., Qadir A., Rehman S.A., Iqubal A., Goh K.W., Govind R., Iqubal M.K., Kesharwani P. (2026). From concept to clinic: Cutting-Edge advances steering the next wave of antibody–drug conjugates innovation. J. Drug Deliv. Sci. Technol..

[283] Cho M., Cervadoro A., Ramirez M.R., Stigliano C., Brazdeikis A., Colvin V.L., Civera P., Key J., Decuzzi P. (2017). Assembly of Iron Oxide Nanocubes for Enhanced Cancer Hyperthermia and Magnetic Resonance Imaging. Nanomaterials.

[284] Zhu C.-B., Carneiro A.M., Dostmann W.R., Hewlett W.A., Blakely R.D. (2005). p38 MAPK Activation Elevates Serotonin Transport Activity via a Trafficking-independent, Protein Phosphatase 2A-dependent Process. J. Biol. Chem..

[285] Han X., Li Y., Zhu X., Zhang P. (2025). A dual chemical-photothermal drug delivery platform inhibits LPS-induced oxidative damage by suppressing the p38 MAPK pathway in cardiomyocytes. Arab. J. Chem..

[286] Tian Y., Lei Y., Wang Y., Lai J., Wang J., Xia F. (2023). Mechanism of multidrug resistance to chemotherapy mediated by P-glycoprotein (Review). Int. J. Oncol..

[287] Raucher D., Dragojevic S., Ryu J. (2018). Macromolecular Drug Carriers for Targeted Glioblastoma Therapy: Preclinical Studies, Challenges, and Future Perspectives. Front. Oncol..

[288] Ashique S., Afzal O., Yasmin S., Hussain A., Altamimi M.A., Webster T.J., Altamimi A.S.A. (2023). Strategic nanocarriers to control neurodegenerative disorders: Concept, challenges, and future perspective. Int. J. Pharm..

[289] Feng J., Tong Y., Zhang Z., He Y. (2025). Case Report: Pathological complete response yet early brain relapse in HER2-positive breast cancer: A case-based review. Front. Immunol..

[290] Wang L., Liang H., Sun J., Liu Y., Li J., Li J., Li J., Yang H. (2019). Bispecific Aptamer Induced Artificial Protein-Pairing: A Strategy for Selective Inhibition of Receptor Function. J. Am. Chem. Soc..

[291] Białeckia P., Adamiak M., Pędziwiatr-Werbicka E., Robaszkiewicz A. (2026). Perspectives of EGFR co-targeting with nanocarriers for anti-TNBC purposes. Genes Dis..

